# Proximity Mapping of Desmosomes Reveals a Striking Shift in Their Molecular Neighborhood Associated With Maturation

**DOI:** 10.1016/j.mcpro.2024.100735

**Published:** 2024-02-10

**Authors:** Judith B. Fülle, Rogerio Alves de Almeida, Craig Lawless, Liam Stockdale, Bian Yanes, E. Birgitte Lane, David R. Garrod, Christoph Ballestrem

**Affiliations:** 1 Wellcome Trust Centre for Cell-Matrix Research, University of Manchester, Manchester, UK; 2 Skin Research Institute of Singapore, Agency of Science Technology and Research (A∗STAR), Singapore, Singapore

**Keywords:** desmosome, intercellular junction, cell-cell adhesion, plakoglobin, plakophilin, desmocollin, epithelial cells, hyper-adhesion, proximitome, BioID, adhesion maturation

## Abstract

Desmosomes are multiprotein adhesion complexes that link intermediate filaments to the plasma membrane, ensuring the mechanical integrity of cells across tissues, but how they participate in the wider signaling network to exert their full function is unclear. To investigate this, we carried out protein proximity mapping using biotinylation (BioID). The combined interactomes of the essential desmosomal proteins desmocollin 2a, plakoglobin, and plakophilin 2a (Pkp2a) in Madin–Darby canine kidney epithelial cells were mapped and their differences and commonalities characterized as desmosome matured from Ca^2+^ dependence to the mature, Ca^2+^-independent, hyper-adhesive state, which predominates in tissues. Results suggest that individual desmosomal proteins have distinct roles in connecting to cellular signaling pathways and that these roles alter substantially when cells change their adhesion state. The data provide further support for a dualistic concept of desmosomes in which the properties of Pkp2a differ from those of the other, more stable proteins. This body of data provides an invaluable resource for the analysis of desmosome function.

## Highlights

• Identification of the proximitomes of canonical desmosomal proteins.
• Comparison of the proximitomes of Ca^2+^-dependent and hyper-adhesive desmosomes.
• A constant molecular neighborhood and changes with the desmosomal adhesion state.
• Insights into the cellular and molecular functions of the proximal proteins are given.

## Graphical Abstract

**Figure:**
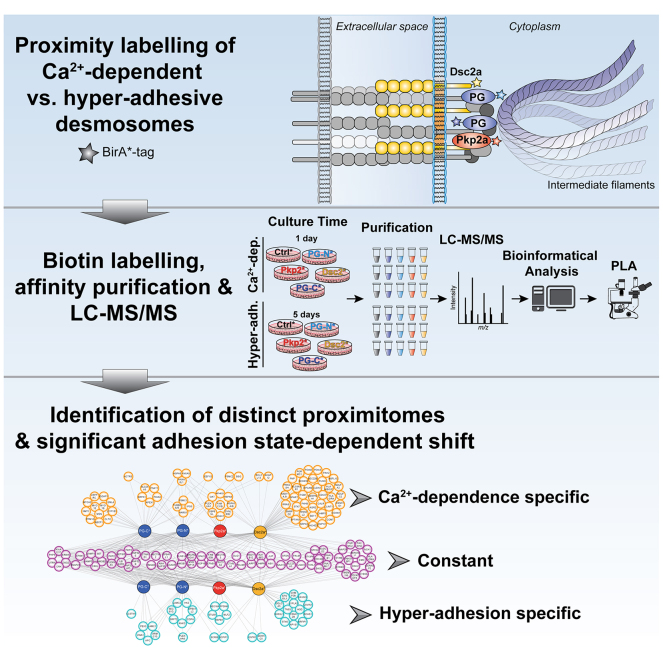

## In Brief

eTOC synopsis: The desmosome proximitome reveals novel associations with potential for desmosome regulation and signaling and shows substantial changes as the junctions mature to hyper-adhesion.

Desmosomes are complex, multiprotein cell–cell junctions providing strong intercellular adhesion and connecting to cytoplasmic intermediate filament (IF) networks. These functions are especially vital for mechanically resilient tissues such as the epidermis and cardiac muscle. The association of impaired desmosome function with severe diseases has revealed that both mechanical resilience and multiple signaling pathways are disrupted in affected tissues (1, 2, 3). However, the underlying mechanisms of abnormal desmosomal function are poorly understood.

The canonical desmosomal complex comprises two kinds of cell adhesion receptors (the desmosomal cadherin [DC] proteins desmogleins (Dsg1–4) and desmocollins [Dsc1–3 a and b]), two adaptor proteins (the armadillo proteins plakophilins [Pkp1–3] and plakoglobin [PG, gene name JUP]), and the plakin protein desmoplakin (DP). DP connects the desmosome junction to the IF network of the cell, forming the desmosome–IF complex, a mechanically resilient tissue-wide scaffolding (1, 4, 5).

During and shortly after assembly in tissue culture, desmosomal adhesion is Ca^2+^-dependent and can be disrupted by the removal of extracellular Ca^2+^. *In vivo*, this default state is seen in early embryonic tissues and wound epidermis (6). Following assembly, Ca^2+^-dependent desmosomes mature, both in culture and *in vivo*, to adopt a more stable Ca^2+^-independent adhesion state referred to as hyper-adhesion, which is the normal condition found in epithelia at homeostasis (6, 7, 8). This maturation process appears characteristic of desmosomes and distinguishes them from adherens junctions (AJ), the other major type of adhesive epithelial junction. The mechanisms by which cells regulate desmosomal adhesion are not fully understood. There is evidence that various reversible posttranslational modifications such as AKT serine/threonine kinase 2-mediated phosphorylation of Pkp1, ribosomal S6 kinases 1 and 2-mediated phosphorylation of Pkp3, and PKCα-mediated phosphorylation of DP are critical for the trafficking of desmosomal proteins and the adhesion state of desmosomes (8, 9, 10, 11, 12, 13, 14, 15, 16, 17). However, PKCα is not essential for desmosome assembly (13). It is also becoming increasingly evident that the cortical actin and the actomyosin networks play a crucial role in desmosome maintenance and *vice versa* (18, 19, 20, 21, 22, 23). However, what consequences the change of desmosomal adhesion state signify is not known.

Desmosomes are remarkably stable structures. They persist even when cells are undergoing mitosis (24), and when epithelial cells separate, desmosomes are torn out of the neighboring cell to be engulfed as whole entities (25). However, overall stability is only part of the story, because the desmosomal complex exhibits dualistic properties, in which DCs, PG, and DP remain quite stably associated with the junctions, at least one protein, Pkp2a, is remarkably dynamic, shuttling in and out of desmosomes to other cellular compartments within seconds (25). This behavior of Pkp2a together with the presence of PG and Pkps in other cellular compartments, including the nucleus, supports a model whereby desmosomes are not simply mediating firm adhesion but also function as signaling platforms (2, 15, 26). Very little is known about how such signaling is organized and how desmosomes connect to signaling networks. It is also unclear how the adhesion state of desmosomes, which is often neglected despite its importance, is regulated and how it affects signaling capacities and pathways.

Here, we have used BioID-coupled mass spectrometry (MS) to study how desmosomal components are embedded in signaling networks (27). To reveal changes in relation to desmosome adhesion states, we generated proximitomes from Dsc2a, PG, and Pkp2a baits with either Ca^2+^-dependent or hyper-adhesive desmosomes. The resulting combined network showed substantial changes in proximal proteins and their possible functions as desmosomes mature, as well as revealing novel partners with potentially novel signaling roles. Moreover, the results reinforce our view of the dualistic nature of desmosomal protein for while Dsc2a and PG share many partners with common functions those of Pkp2a differ considerably. The results expand our view of desmosomes and their roles beyond mechanically resilient cell–cell junctions.

## Experimental Procedures

### Reagents

Primary antibodies used at the indicated dilutions were rabbit α-DP (used at 1:200; A303-356A; Bethyl Laboratories); mouse α-Myc (used at 1:100 for immunostainings and 1:1000 for Western blottings; clone 9B11; Cell Signaling Technology); rabbit α-γ-catenin (PG; used at 1:200 for colocalization stainings and 1:1000 for proximity ligation assay (PLA); ab15153; Abcam); rabbit α-KIAA1217 (used at 1:500 for colocalization stainings and PLA; PA5-85468; Invitrogen; kindly provided by Tobias Zech, University of Liverpool); mouse α-Desmocollin 2/3 (used at 1:200 for colocalization stainings and PLA; clone 7G6; 32-6200; Invitrogen); mouse α-p120 catenin (used at 1:400 for colocalization stainings and 1:1000 for PLA; 612536; BD Transduction Laboratories), and mouse α-Lamin A/C (used at 1:200 for colocalization stainings and PLA; mab636; MA3-1000; Invitrogen). Secondary antibodies conjugated to Alexa Fluor 488 or 647 were from Jackson ImmunoResearch and used at 1:500 for immunostainings and α-mouse Alexa Fluor 680 from Thermo Fisher Scientific was used at 1:5000 from Western blotting. Alexa Flour 680–conjugated streptavidin was used at 1:500 dilution for immunostainings and Alexa Fluor 800 was used at 1:1000 for Western blotting (both from Thermo Fisher Scientific). 4′,6-diamidino-2-phenylindole readymade solution (Sigma) was used at a concentration of 1 μg/ml for immunostainings.

### Cell Culture

Madin–Darby canine kidney II cells (MDCK; ECACC) (28) were cultured at 37 °C in 5% humidified CO_2_ in high glucose Dulbecco’s Modified Eagle Medium (Sigma), supplemented with 10% (v/v) fetal calf serum (Gibco) and 100 U/ml penicillin and 100 μg/ml streptomycin (P/S; Gibco).

### Cloning

To generate constructs containing BirA-myc, cloning was performed using Gibson Assembly Cloning Kit #EE5510S, New England Biolabs (NEB) according to the manufacturer’s protocol. Vectors were linearised with single restriction endonucleases obtained by NEB, and fragments were generated by PCR using Phusion High-Fidelity Polymerase (#M0530L, NEB) using 35 cycles and 60 °C annealing temperature. All primers were designed using SnapGene (GSL Biotech LLC) and were synthesized by Eurofins Genomics (Supplemental Table S5). BioID vectors with the BirA(R118G) tag, pCDH-BirA-myc, and pCDNA3.1-myc-BioID (Addgene #35700; (29)) were gifts from A. Gilmore (University of Manchester) and C. van Itallie (NIH). To generate puro-BirA-myc containing expression vectors, BirA-myc was obtained by PCR using pCDH-BirA-myc as a template with primers BirA-myc.for and BirA-myc.rev and cloned *via* NcoI site into a custom-made vector pSF (#0G394R1, Oxford Genetics), which was modified to have an eukaryotic translation initiation factor 1 promoter and a puromycin selection marker. To clone myc-BirA, pCDNA.31-myc-BioID and primers myc-BirA.for and myc-BirA.rev were used and cloned into pSF *via* the NcoI site. Plasmid DNA–containing desmosome genes were purchased from Addgene, [Dsc2a #32233 (30), PG #32228 (31), and Pkp2a #32230 (32)] and used as templates for PCRs to obtain their ORFs (Supplemental Table S5). To construct puro-Dsc2a-BirA-myc and puro-PG-BirA-myc the primers Dsc2a.for and Dsc2a.rev, PG-BirA.for and PG-BirA.rev were used, respectively, for both constructs, the fragments were cloned into the HindIII site. Puro-myc-BirA-PG was cloned into puro-myc-BirA *via* BbvCI site using primers BirA-PG.for and BirA-PG.rev, and Pkp2a was amplified using Pkp.for and Pkp.rev and cloned into puro-myc-BirA *via* BbvCI site creating puro-myc-BirA-Pkp. All ORF sequences were confirmed by Sanger sequencing performed by Eurofins.

### Generation of Stable Cell Lines

Cells were transfected using Lipofectamine LTX transfection reagent, according to the manufacturer’s instructions (Invitrogen). To generate stable cell lines, transfected MDCK cells were selected using 2 μg/ml puromycin (Thermo Fisher Scientific) with medium changes every 2 days for 10 days.

### Immunofluorescence Microscopy

Cells were fixed with either 100% ice-cold methanol or 4% (w/v) paraformaldehyde in PBS, followed by an additional permeabilization step with 0.1% (v/v) Triton X-100 + 1% (w/v) bovine serum albumin in PBS. Antibodies were diluted in 1% bovine serum albumin and added to the cells for 1 h. Images were acquired on a Delta Vision microscope (Applied Precision) with a 60 × /1.42 Plan Apo N (Oil) objective and a Sedat Quad filter set, with images collected using a Retiga R6 (Q-Imaging) camera and processed using the FIJI ImageJ software (version 1.53 g; https://fiji.sc/).

### *In situ* PLA

The Duolink *in situ* Red Starter Kit (Sigma-Aldrich, DUO92101) was used in the PLA according to the manufacturer’s instructions and following previously published methods (33). Briefly, MDCK cells were fixed, permeabilized, and incubated with primary antibodies as described in “Immunofluorescence microscopy.” After multiple washes, cells were incubated with PLA probes (α-Rabbit PLUS, α-Mouse MINUS) and successively incubated with ligation solution and amplification solution. Cells were counterstained with a pair of secondary antibodies conjugated with Alexa Fluor 488 or 647 to provide spatial data and imaged as before. Images were analyzed using FIJI ImageJ software (version 1.53gg). The total number of PLA signals per cell and the number of signals within a 1 μm range around the plasma membrane (junctions) were counted. The cytoplasmic signals were counted and the ratio of junctional to cytoplasmic signals was plotted using Microsoft Excel and GraphPad Prism.

### Western Blotting

For western blotting, SDS-PAGE gels (Novex, Thermo Fisher Scientific) were blotted on nitrocellulose membranes (Immobilon) and incubated with the respective antibodies. Secondary antibodies were detected *via* an Odyssey IR imaging system (LI-COR). Blots were analyzed using FIJI ImageJ software (version 1.53 g).

### Proximity Biotinylation and Affinity Purification

To mediate proximity biotinylation, cells expressing BirA-myc constructs were seeded at confluent density (1.35 × 10^5^ cells/cm^2^) onto 15-cm plastic cell culture dishes for 8 h for Ca^2+^-dependent desmosome formation or 4 days and 8 h for hyper-adhesive desmosome maturation, and then incubated in medium supplemented with 100 μM biotin (#B20656, Life Technologies) for 16 h (*i.e.*, total culture time was 1 day or 5 days). Biotinylated proteins were affinity purified, following a protocol adapted from Chastney *et al.* (34). Cell lysis was performed in a 4 °C cold room. The cells were washed thrice with cold 15 ml PBS (without CaCl_2_ and MgCl_2_) and lysed with 1.2 ml lysis buffer (50 mM Tris pH 7.4, 500 mM NaCl, 0.4% [wt/vol] SDS, 5 mM EDTA, 1 mM DTT, and 1x cOmplete Protease inhibitor cocktail, Roche) for 20 min on a rocker at 4 °C. Cells were scraped, transferred in 5 ml tubes (Eppendorf), and 480 μl Triton-X-100 (2% [vol/vol] final concentration) was added. Samples were further lysed on ice by three sonication steps for 30 s each using a Vibra-Cell VCX750 (Sonics) at 20% power. Two hundred twenty microliters of cold 1M Tris–HCl pH 7.4 (20 mM final concentration) was added and the samples were centrifuged for 20 min at full speed (16,000 rpm) at 4 °C. The supernatant was rotated with 50 μl MagReSyn streptavidin beads (#MR-STV0101, 2B Scientific), which were equilibrated with lysis buffer, at 4 °C overnight. Samples were transferred into 1.5 ml centrifuge tubes and placed into a magnetic rack. Beads were washed twice with 1 ml wash buffer 1 (2% [wt/vol] SDS], once with 1 ml wash buffer 2 (0.1% [wt/vol] deoxycholate, 1% [wt/vol] Trition X-100, 500 mM NaCl, 1 mM EDTA, 50 mM Hepes pH 7.4), and once with 1 ml wash buffer 3 (250 mM LiCl_2_, 0.5% [wt/vol] NP-40, 0.5% [wt/vol] deoxycholate, 1 mM EDTA, 10 mM Tris pH 8.1). Each washing step was performed for 8 min at room temperature using rotation. Proteins were eluted in 100 μl 2x reducing sample buffer (2% [wt/vol] SDS, 12% [wt/vol] sucrose, 0.004% [wt/vol] bromphenol blue, 50 mM Tris/HCl pH 6.8, 10% [wt/vol] 2-mercaptoethanol (or DTT), and 10 mM biotin) for 10 min at 90 °C with mixing every 2 min. Biotinylation of proteins was confirmed using Western blotting and equal loading *via* Ponceau S staining. Samples were then analyzed using LC-MS/MS.

To prepare samples for MS, 20 μl of eluted proteins were briefly run on SDS-PAGE (4 min at 160 V, 10% SDS gel [#NP0301, Invitrogen]), stained with SimplyBlue SafeStain (#LC6065, Invitrogen) for 1 h and washed with ddH_2_O four times for 5 min each. For protein digestion, bands of interest were excised from the gel and dehydrated using acetonitrile, followed by vacuum centrifugation. Dried gel pieces were reduced with 10 mM DTT and alkylated with 55 mM iodoacetamide. Gel pieces were then washed alternately with 25 mM ammonium bicarbonate, followed by acetonitrile. This was repeated, and the gel pieces were dried by vacuum centrifugation. Samples were digested with trypsin overnight at 37 °C.

### MS Data Acquisition

Digested samples were analyzed by LC-MS/MS using an UltiMate 3000 Rapid Separation LC (Dionex Corporation) coupled to an Orbitrap Exploris 480 (Thermo Fisher Scientific) mass spectrometer. Mobile phase A was 0.1% formic acid in water and mobile phase B was 0.1% formic acid in acetonitrile and the column used was a 250 mm × 75 μm i.d. 1.7 μM nE MZ PST CSH130 C18, analytical column (Waters). A 2 μl aliquot of the sample was transferred to a 5 μl loop and loaded onto the column at a flow of 300 nl/min for 8 min, which then ramped to 5% B in 2 min. Peptides were separated using a gradient that went from 5% to 21% B in 44 min, and then from 21% to 31% B in 7 min and finally from 31% B to 65% B in 1 min. The column was washed at 65% B for 4 min before dropping to 1% B in 1 min and re-equilibration for a further 8 min. Data were acquired in a data-dependent manner using a fixed cycle time of 1.5 s, an expected peak width of 15 s and a default charge state of 2. Full MS data were acquired in positive mode over a scan range of 300 to 1750 Th, with a resolution of 120,000, a normalized automatic gain control target of 300% and a max fill time of 25 mS for a single microscan. Fragmentation data were obtained from signals with a charge state of +2, +3, or +4 and an intensity over 5000, and they were dynamically excluded from further analysis for a period of 15 s after a single acquisition within a 10 ppm window. Fragmentation spectra were acquired with a resolution of 15,000 with a normalized collision energy of 30%, a normalized automatic gain control target of 300%, first mass of 110 Th, and a max fill time of 25 mS for a single microscan. All data were collected in profile mode.

The MS proteomics data have been deposited to the ProteomeXchange Consortium *via* the PRIDE (35) partner repository with the dataset identifier PXD037933.

All raw data were processed using MaxQuant software (v 1.6.10.43; https://maxquant.org) (Supplemental Tables S1 and S2 and ProteomeXchange (https://proteomexchange.org) (36). Spectra were searched against the canine (*Canis lupus familiaris* Taxon ID 9615) proteome obtained from UniProt (45,351 proteome sequences, November 2020). All annotated spectra can be accessed through PRIDE and in addition the specific annotated spectra for proteins identified based on a single unique peptide can accessed *via* https://msviewer.ucsf.edu/prospector/cgi-bin/mssearch.cgi?report_title=MS-Viewer&search_key=70wjnsvdlz&search_name=msviewer. Biotinylation of lysine, methionine oxidation, and N-terminal acetylation were set as a variable modification, with carbamidomethylation of cysteine as a fixed modification. Precursor tolerance was set at 20 ppm and 4.5 ppm, for the first and main search, respectively, with tandem mass spectrometry tolerance set at 20 ppm and up to two missed cleavages were allowed. The false discovery rate of peptide-to-spectrum match and protein was set at 0.01 and “match between runs” was enabled.

### Bioinformatical Analyses

Protein intensities were exported from MaxQuant, normalized by median-centering, and analyzed through SAINTexpress software (v3.6.3; https://saint-apms.sourceforge.net) (37). Bait samples were grouped and analyzed against their respective condition-specific control samples. In total, we have identified 3607 proteins under calcium-dependent conditions and 4050 proteins under hyper-adhesive conditions that were enriched in comparison to the BirA-myc control, which can all be accessed through PRIDE. Significant proximal bait–prey interactions were taken using a Bayesian false discovery rate (BFDR) threshold of 0.05.

Before any Gene Ontology (GO) analysis, human orthologs were obtained, where possible, for all quantified canine proteins using the Ensembl BioMart Service (Ensembl release 102) (38, 39) and manual curation using the PANTHER database (v 16) (40). For seven protein hits, we identified protein groups which cannot be distinguished with the current MS methodology. These hits comprised HLA-F/HLA-A/HLA-B/HLA-C/, RAB11A/RAB11B, acidic nuclear phosphoprotein (ANP)32D/ANP32A, TUBB2B/TUBB2A, HSPA1B/HSPA1A, DYNLRB2/MAP1LC3A, DNAJB7/DNAJB6, which were included separately for network and GO analyses. All subsequent GO functional analyses were then performed in the R environment (41) using the R package ClusterProfiler (v 4.0.3) (42) against human GO annotations.

For hierarchical clustering, a list of prey proteins that were significant at BFDR ≤0.05 for any bait in either condition was taken. Hierarchical clustering was then carried out on the list of preys based on the Jaccard distance of significant (BFDR ≤0.05) and nonsignificant (BFDR >0.05) interactions across all baits and both conditions. A heatmap was rendered to visualize the prey clusters, overlaying the log_2_ fold-change enrichment over BirA-myc control in each bait condition.

Network visualization and analysis were performed using Cytoscape (v 3.9.0; https://cytoscape.org) (43). Network analysis was performed using the stringApp plugin in Cytoscape, which is based on the STRING (search tool for the retrieval of interacting genes/proteins) database (v 11.5 accessed October 2021) (44, 45). STRING scores of ≥0.7 and <0.7 were distinguished and edges were discretely mapped accordingly.

Protein–protein interactions were compared with the biological general repository for interaction datasets for DSC2, PKP2, and JUP (BioGRID v 4.4) (46) (available from https://thebiogrid.org/). Protein functions in Figure 6 were annotated manually based on the primary literature and the Human Protein Atlas (47) (available from http://www.proteinatlas.org).

Graphing was performed using GraphPad Prism (v 9.2.0) (GraphPad Software, www.graphpad.com). Venn diagrams were generated with the help of BioVenn (48). Figures were assembled in Adobe Illustrator (v 26.0.1).

### Experimental Design and Statistical Rationale

For BioID experiment, biological triplicates per bait (Dsc2a∗, PG-N∗, PG-C∗, Pkp2a∗, BirA-myc control) and condition (Ca^2+^-dependent or hyper-adhesive) were employed. A total of 30 samples were analyzed by LC-MS/MS. Statistical scoring was performed against three negative controls (BirA-myc) per condition using SAINTexpress as described in detail in the section bioinformatical analyses. Significant proximal bait–prey interactions were taken using a BFDR threshold of 0.05. Imaging and western blot data are representative of results from at least three independent experiments.

## Results and Discussion

### Proximitome of Core Desmosome Components

**Fig. 1:**
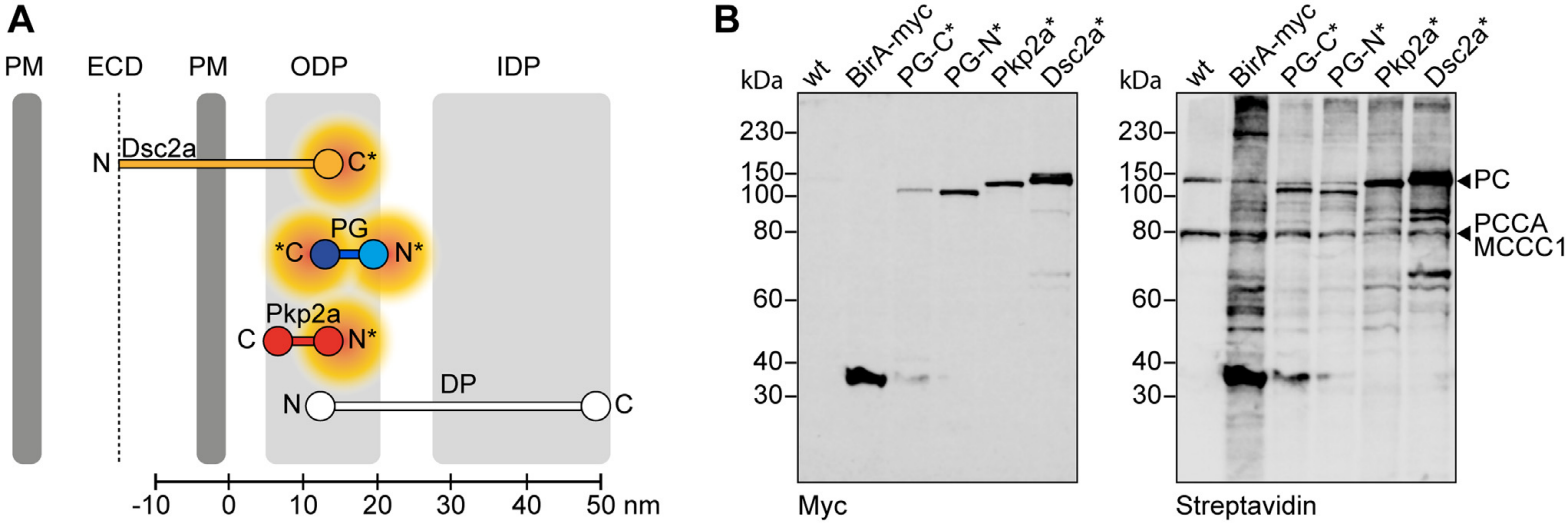
**BioID constructs to generate desmosome proximitome.***A*, schematic representation of the biotin ligase (BirA)-tagged desmosomal constructs, desmocollin 2a (Dsc2a, *yellow*), plakoglobin (PG, *blue*), and plakophilin 2a (Pkp2a, *red*) within the desmosomal molecular map based on work from North *et al.* (112). The *asterisk* indicates the terminus, which was tagged with BirA-myc (C terminus) or myc-BirA (N terminus). The *orange cloud* indicates the estimated radius of biotinylation by the biotin ligase. *B*, Western blot of MDCK cells stably expressing BirA-myc as control, PG-BirA-myc (PG-C∗), myc-BirA-PG (PG-N∗), myc-BirA-Pkp2a (Pkp2a∗), or Dsc2a-BirA-myc (Dsc2a∗) and parental WT cells as a negative control. The cells were cultured confluent for 24 h, including 16 h with 100 μM biotin before lysis and separation of biotinylated proteins with streptavidin beads. The blot was probed with a mouse-α-myc antibody to detect expression constructs and fluorescently conjugated streptavidin for biotinylated proteins. Note the bands in WT cells correspond with naturally biotinylated mitochondrial carboxylases, including pyruvate carboxylase (PC), methylcrotonoyl-CoA carboxylase subunit alpha (MCCC1), and propionyl-CoA carboxylase alpha chain (PCCA). Blot is representative of three biological replicates. DP, desmoplakin; ECD, extracellular core domain; IDP, inner dense plaque; ODP, outer dense plaque; PM, plasma membrane.

To investigate the proximitome of desmosomes, BioID was carried out (Fig. 1*A*). We selected Dsc2a, PG, and Pkp2a as BioID baits. The proteins were cloned into a custom-made vector containing BirA(R118G)-myc (indicated by an asterisk) and stably expressed in the simple epithelial cell line MDCK. Dsc2a was tagged on its cytoplasmic C terminus (Dsc2a-BirA-myc, Dsc2a∗), PG on both the N- (myc-BirA-PG, PG-N∗) and C-termini (PG-BirA-myc, PG-C∗), and Pkp2a on its N terminus (myc-BirA-Pkp2a, Pkp2a∗). Tagging of the C terminus of Pkp2a was dismissed as it interfered with localization to desmosomes (data not shown), confirming findings that the C terminus of Pkps, in particular of Pkp1, is required for recruitment to the plasma membrane (49). As controls, we generated MDCK cells stably expressing BirA-myc alone (27).

Western blotting showed expression of all constructs with expected mobilities: 36.5 kDa for BirA-myc, 118.5 kDa for PG-N∗ and PG-C∗, 134 kDa for Pkp2a∗, and 129.5 kDa for Dsc2a-BirA-myc (Fig. 1*B*, left blot). Incubation with fluorescently conjugated streptavidin confirmed the biotinylation capacities of the constructs with the most intense bands representing the BioID baits and other bands biotinylated preys (Fig. 1*B*, right blot). Naturally biotinylated mitochondrial carboxylases and ribosomal proteins, which can be biotinylated during protein translation, were excluded from further MS analyses (Fig. 1*B*, Supplemental Tables S1 and S2; (27)). All baits colocalized with DP-positive structures (Supplemental Fig. S1*A*, left panel), showing that the BirA tag did not hinder subcellular targeting to desmosomes. Staining for biotinylated proteins revealed distinct distributions in close proximity to the bait proteins, except for a stronger nuclear signal in the BirA-myc–expressing cells (Supplemental Fig. S1*A*, right panel). These data demonstrated that BirA-tagged desmosomal proteins localized at the expected cell–cell junction sites, where they biotinylate their neighborhood.

When desmosomes first form between MDCK cells they are Ca^2+^-dependent but the majority become hyper-adhesive within 5 days of confluent culture (8). To determine proximal interactors of each desmosomal BioID bait depending on the desmosomal adhesion state, MDCK cells stably expressing BioID constructs were cultured confluent for 1 day (Ca^2+^-dependent condition) or 5 days (hyper-adhesive condition). The expression of the BioID constructs has no significant effect on the acquisition of hyper-adhesion (Supplemental Fig. S1, *B* and *C*).

**Fig. 2:**
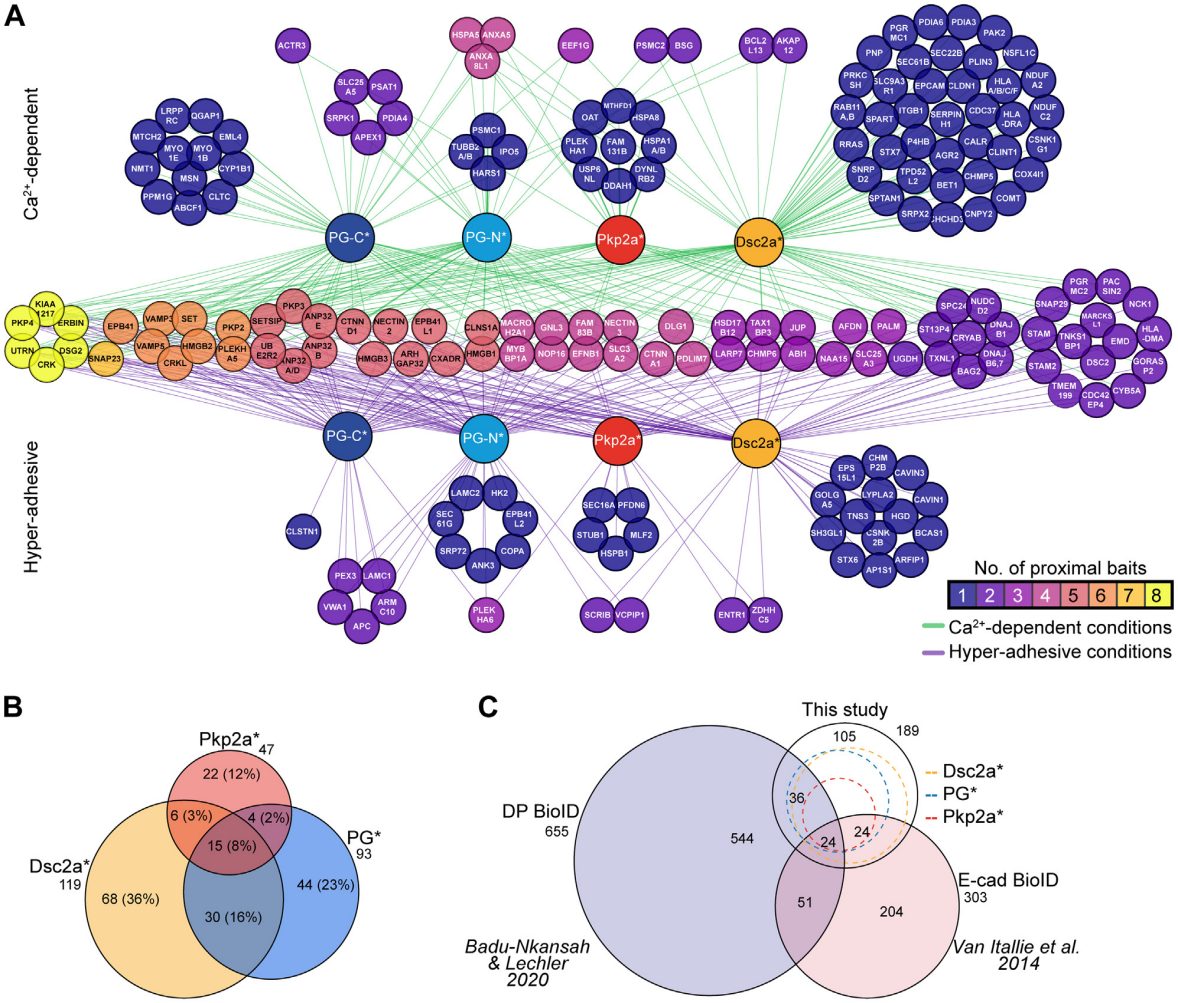
**Overview and gene ontology analysis of the partial proximitome of Ca**^**2+**^**-dependent and hyper-adhesive desmosomes.***A*, network of proximal proteins (gene names of human orthologs) to BirA-myc–tagged desmosomal constructs, plakoglobin-BirA-myc (PG-C∗, *dark blue*), myc-BirA-plakoglobin (PG-N∗, *light blue*), myc-BirA-plakophilin 2a (Pkp2a∗, *red*), and desmocollin 2a (Dsc2a∗, *yellow*). Proximal proteins on the *top* are those identified in samples of MDCK cells cultured confluently for 1 day and thus with Ca^2+^-dependent desmosomes (*green edges*), those on the bottom were found in samples of cells cultured confluently for 5 days and thus hyper-adhesive desmosomes (*magenta edges*) and those in the *middle* were detected in both samples, all with a high-confidence cut-off of BFDR ≤0.05. Proximal prey proteins are presented using a color-coded scale, indicating the number of proximal baits. *B*, area-proportional Venn diagram of proximal proteins to Dsc2a∗, Pkp2a∗, and PG∗ (combined PG-C∗ and PG-N∗). *C*, area-proportional *Venn diagram* comparing the prey proteins of Dsc2a∗, PG∗, and Pkp2a∗ to those found in proximity to desmoplakin (DP; (50)) and the adherens junction receptor E-cadherin (E-cad; (52)). Number of proximal proteins is indicated. BFDR, Bayesian false discovery rate; Dsc, desmocollin; PG, plakoglobin; Pkp, plakophilin.

The proximal proteins (preys) were identified using label-free quantitative MS and analysis of the raw data was performed using ion intensity-based quantification with MaxQuant against the *C. lupus familiaris* proteome. We used SAINTexpress to identify high-confidence bait–prey proximal interactions with BirA-myc as a negative control. To predict “true” proximity interactors, a stringent BFDR of ≤0.05 was employed and the respective human orthologs were obtained for subsequent analyses. This resulted in a dataset with a total of 189 proximal proteins, which included a combination of canonical desmosomal proteins, desmosome-associated proteins, and proximal proteins of the BioID baits in ostensibly more distal subcellular locations (Fig. 2*A* and Supplemental Table S3).

We integrated the eight individual datasets of Dsc2a∗, PG-N∗, PG-C∗, and Pkp2a∗ from both Ca^2+^-dependent and hyper-adhesive conditions into a single network to generate a desmosome proximitome consisting of a total of 259 protein–protein interactions (Fig. 2*A*). The overlap between the proximitomes of the combined data for the various baits is shown in Figure 2*B*. The network illustrates both the distinct and common proximal proteins to each bait in relation to the desmosomal adhesion state and is organized as follows. The preys in the horizontal middle of the scheme associated with the baits in both desmosomal adhesion states. These “constant” proteins represent 39% (74 proteins) of the total. The color index indicates how many of the baits shared the preys, with the proteins on the left (yellow) shared by all baits and those on the right shared only one of the baits. The proteins on the upper side of the schematic (78 or 41%) were found only in proximity during Ca^2+^ dependence, whereas those at the bottom (37 or 20%) were uniquely associated during hyper-adhesion. The large pools of proteins uniquely associated with particular adhesion states reveal a dramatic change in the neighborhood of the desmosomal proteins as well as a substantial reduction in their number during desmosome maturation (152 proteins enriched in Ca^2+^-dependent samples to 111 proteins in hyper-adhesive samples).

When comparing the datasets for the different baits, more than half of the proximal proteins (71%, 134 proteins) were unique to a specific bait, whereas 55 proteins (29%) were identified by at least two different desmosomal baits and may therefore represent shared pivotal desmosomal components (Fig. 2*B* and Supplemental Table S3).

Comparison with the recently published DP proximitome in mouse keratinocytes revealed that 32% (60 proteins) of proximal proteins were shared with our Dsc2a, PG, and Pkp2a bait datasets (Fig. 2*C* and Supplemental Fig. S2*A*). These included known desmosomal components, such as Pkp3 and Dsg2, as well as other reported interactors such as erbin and the Crk family of adaptor proteins (50, 51). Among the interactors in the DP proximitome were keratins, IF proteins that were not significantly enriched in our datasets in relation to the BirA-myc control, reflecting the specific function of DP in connecting IFs to desmosomes. Further comparison with BioID data comprising proximal proteins of the AJ protein E-cadherin in MDCK cells (52) revealed 48 mutual proteins (25%) with our datasets (Fig. 2*C* and Supplemental Fig. S2*A*). The majority of proteins shared with E-cadherin were identified in association with Dsc2a∗ (39/48). Uniquely identified under Ca^2+^-dependent conditions (newly formed desmosomes), nine of these Dsc2a∗-proximal proteins, including the endosomal protein syntaxin 7 and the endoplasmic reticulum channel protein sec61 translocon β (SEC61B), are involved in protein trafficking and are published Dsc2 interactors (BioGRID, (53)). This may suggest that Dsc2a and E-cadherin follow similar routes during junction formation. The reduction of proximal proteins shared with E-cadherin as desmosomes mature to hyper-adhesion from 26 (eight found solely under Ca^2+^-dependent conditions) to 22 (four unique proteins under hyper-adhesive conditions) is in line with current models of desmosome assembly in conjunction with AJ formation and their spatial separation as they mature (54, 55).

### Functional Enrichment Analysis of Biotinylated Proteins

**Fig. 3:**
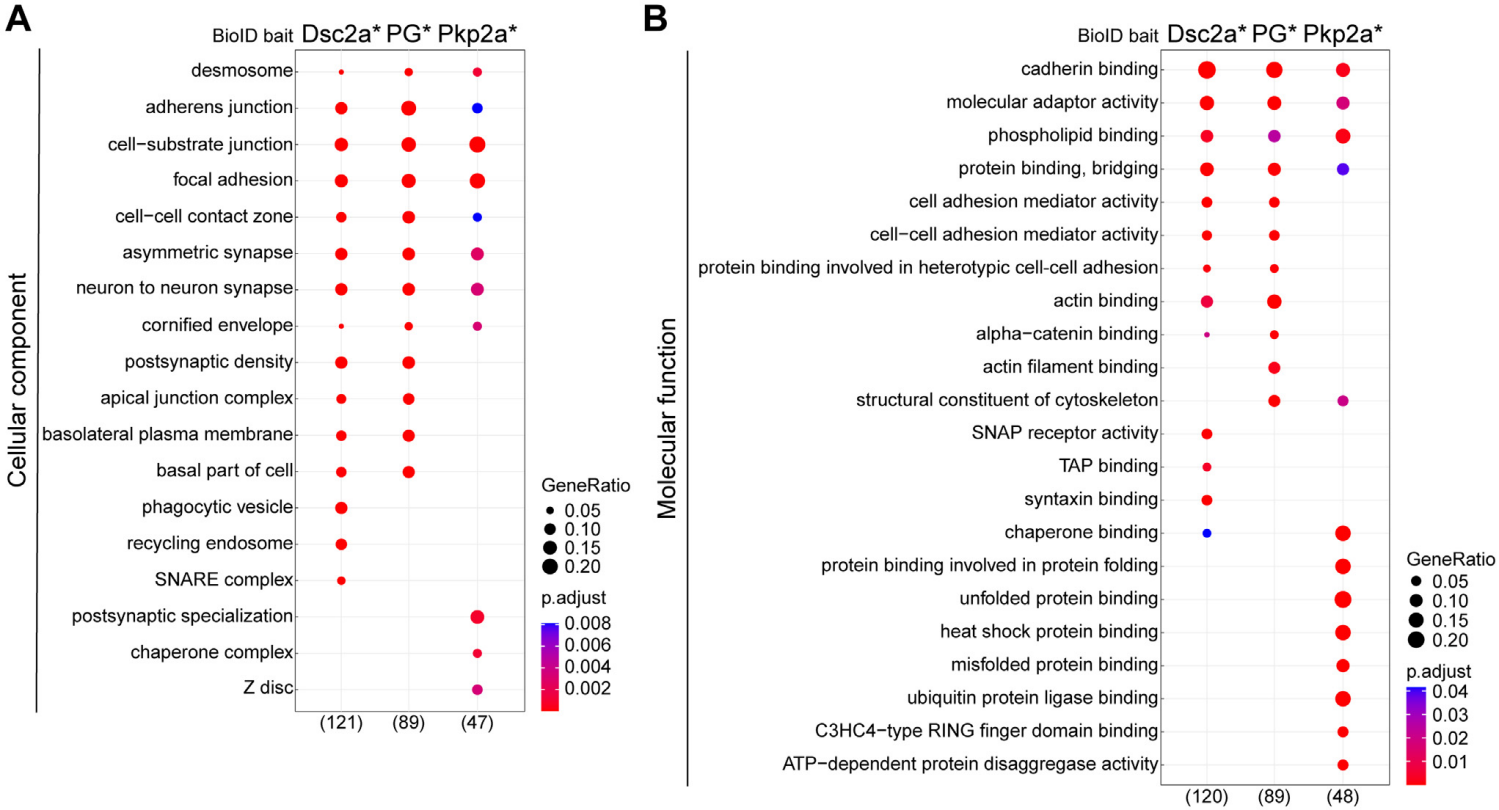
**Ontological analysis of desmosomal proximitomes.** Functional enrichment analysis of proteins identified by BirA-myc–tagged desmocollin 2a (Dsc2a∗), plakoglobin (PG∗; merged data plakoglobin-BirA-myc and myc-BirA-plakoglobin data), or plakophilin 2a (Pkp2a∗) BFDR ≤0.05. The top ten overrepresented GO terms under cellular component (*A*) or molecular function (*B*) category are shown. The number of annotated proteins is shown in *brackets*. (Note: seven protein hits encompassed protein groups of which all members were included for GO analysis because we could not distinguish between different isoforms. For details see Experimental Procedures). p.adjust, adjusted *p* value. GeneRatio, proportion of total proteins identified in each GO term. BFDR, Bayesian false discovery rate; GO, Gene Ontology.

To gain unbiased insight into the diverse functions of the proximity interactors, overrepresentation analysis of the combined proximitomes of both adhesion states was carried out (Fig. 3 and Supplemental Fig. S2*B*, Supplemental Table S4). This allowed investigation into whether the BioID datasets associated with particular cellular components, molecular functions, or broader biological functions as represented by current GO annotations are statistically overrepresented among the proximal proteins. A striking observation to emerge from all three analyses was the great similarity of the proximitomes of Dsc2a∗ and PG∗, contrasting with the substantially distinct Pkp2a∗ proximitome.

Cellular component analysis revealed enrichment of terms related to cell–cell junctions, including “desmosome,” “AJs,” and “apical junction complex” (Fig. 3*A*). Cell-extracellular matrix adhesion terms were also identified (“focal adhesion” and “cell-substrate junction”), possibly reflecting miscellaneous targeting of baits and/or shared components and regulatory pathways as reported for Pkp2, integrin β1, and p120-catenin (p120ctn, CTNND1) (56, 57).

Similarly, analysis of molecular functions demonstrated some common terms between the datasets of all baits including “cadherin binding,” “molecular adaptor activity,” “protein binding, bridging,” and “phospholipid binding,” which correspond with desmosomes being multiprotein complexes associated with the phospholipid plasma membrane (Fig. 3*B*). The majority of molecular function terms for Pkp2a∗ were distinct from those of Dsc2a∗ and PG∗, suggesting a separate signaling role for Pkp2a. Most of the preys based on GO annotations associated with the appropriate folding of proteins include chaperones and heat shock proteins (HSPs) that are further analyzed and discussed below.

In conclusion, the two types of dynamic behavior of desmosomal components that we recently demonstrated (25) are reflected in the divergent nature of their proximitomes, with those of the two stable components Dsc2a and PG being similar in their molecular neighborhood while that of the much more dynamic Pkp2a is markedly distinct.

### Maturation Analysis Outlines Neighborhood Differences Between Desmosomal Components and Shows Substantial Changes as Desmosomes Become Hyper-Adhesive

**Fig. 4:**
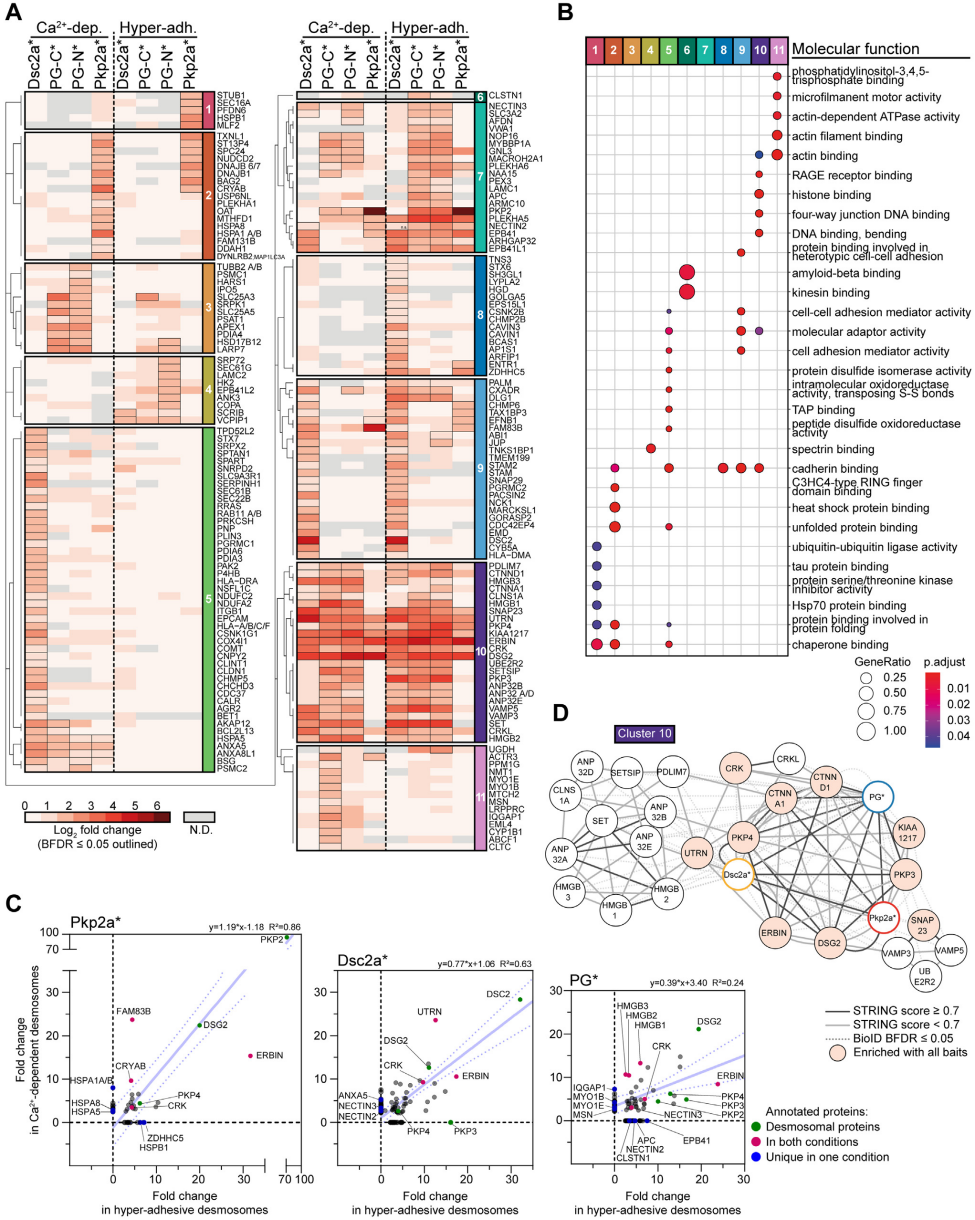
**Differences in the proximitome of Ca**^**2+**^**-dependent and hyper-adhesive desmosomes revealed by hierarchical clustering.***A*, hierarchical clustering was performed on the 189 proteins identified in the partial desmosomal proximitome and displayed as a heatmap. Prey proteins were clustered based on the Jaccard distance of significant (BFDR ≤0.05) and nonsignificant (BFDR >0.05) interactions across all baits that is desmocollin 2a-BirA-myc (Dsc2a∗), myc-BirA-plakoglobin (PG-N∗), plakoglobin-BirA-myc (PG-C∗), myc-BirA-plakophilin 2a (Pkp2a∗) and both conditions that is 1 or 5 days confluently cultured MDCK cells stably expressing BioID baits thus with either Ca^2+^-dependent (Ca^2+^-dep.) or hyper-adhesive desmosomes (Hyper-adh.), respectively. Log2 fold change enrichment over BirA-myc control of prey proteins is displayed as a heatmap. Dendrograms were split to identify clusters of preys (1–11). N.D. = not detected. *B*, GO enrichment analysis of the clusters in *A*. The *top* five overrepresented terms under the molecular function category per cluster are shown. *C*, network analysis of bait–prey interactions of cluster 10 in *A*. Nodes of bait proteins are color codes with Pkp2a∗ in *red*, Dsc2a∗ in *yellow* and PG∗ (merged PG-N∗ and PG-C∗) in *blue*. Nodes of prey proteins are shaded in *light red* when they were present in all bait datasets. Edges indicate protein–protein interactions: *solid dark gray lines* indicate a STRING score ≥0.7; *solid light gray lines* a STRING score below 0.7; and *dotted red lines* BioID proximity with a BDFR ≤0.05 presented in this study. *D*, scatter analysis highlighting changes of prey proteins during desmosome maturation. *Scatter plots* showing the relationship of prey proteins in cells with either Ca^2+^-dependent or hyper-adhesive desmosomes with either Dsc2a∗, Pkp2a∗, or PG∗ as a bait (SAINT fold change enrichment over BirA-myc control). The 95% confidence interval of the linear regression line is displayed as *dotted confidence band*. BFDR, Bayesian false discovery rate; GO, Gene Ontology; MDCK, Madin–Darby canine kidney II cell.

To gain deeper insight into the possible functional significance of the proximitome during the switch in adhesion states, we performed two-dimensional hierarchical clustering and GO enrichment analyses of the clusters of prey proteins (Fig. 4, *A* and *B*; Supplemental Fig. S3*A*). Such hierarchical clustering helped us to separate the data into clusters of similarity related to the adhesion state of the desmosomes. This resulted in 11 clusters, which fell into three categories with respect to desmosome maturation from Ca^2+^ dependence to hyper-adhesion: those that are present throughout, the “constant” proteins (clusters 7, 9, and 10); those that are present exclusively during the Ca^2+^-dependent phase (clusters 3, 5, 11, and a slight bias in cluster 2) and those that are present exclusively during the hyper-adhesive phase (clusters 1, 4, 6, and 8). Moreover, except for cluster 10, the clusters mostly focused on specific desmosomal baits: clusters 3, 4, 6, 7, and 11 were focused mainly on PG∗, clusters 5, 8, and 9 mainly on Dsc2a∗, and clusters 1 and 2 mainly on Pkp2a∗.

To determine how the proximal proteins to PG∗ were shared between its N and C termini, we also generated a heatmap following hierarchical clustering of the datasets at both adhesion states for PG-N∗ and PG-C∗ (Supplemental Fig. S3, *B* and *C*). Thirty nine (42%) of the total 93 proteins were detected by both PG-N∗ and PG-C∗ irrespective of the desmosomal adhesion state (Supplemental Fig. S2*C*). This substantial overlap may be because the range of biotinylation of BirA(R118G) probably covers the central armadillo domain of PG, which binds both DCs (armadillo 1–3), Pkps, and DP and other shared proximal interactors (58).

While some proteins were detected irrespective of the desmosomal adhesion state and thus could be classified as “constant”, it is clear from the heatmaps that many of these exhibited stoichiometric changes during the maturation process (Fig. 4*A* and Supplemental Fig. S3*B*). To examine this possibility in more detail, we generated scatter plots that show the quantitative relationships of prey proteins depending on the adhesion state (Fig 4*C* and Supplemental Fig. S3*C*). These analyses indicate that while some properties of desmosomes persist in all states, others change substantially with the adhesion status of the junction.

To gain further information about how our experimental data overlay with existing databases, we added STRING-based interaction networks to each cluster (Fig. 4*D* and Supplemental Fig. S4) (45). We have selected some of these networks for further discussion below; others will serve as undiscussed resources awaiting future analysis.

### Constant Proteins: Some Novel Interactions and Functional Insights

Proteins that are present throughout the maturation process are likely to be continuously involved in desmosome function. Cluster 10 contains proteins that were observed proximal to all baits (Fig. 4*D*) with the fewest changes between desmosomal adhesion states; cluster 9 proteins were mostly continuously present close to Dsc2a∗, and cluster 7 proteins were enriched proximal to PG∗ in both conditions (Supplemental Fig. S4). These clusters comprised canonical desmosomal proteins (PKP3, PKP4 and DSG2; cluster 10), proteins of other cell–cell adhesion complexes such as the nectin 2 and 3 (cluster 7) and α- and p120-catenin (CTNNA1 and CTNND1; cluster 10) of adherens and gap junctions and surprising lesser-known proteins such as KIAA1217 (cluster 10). KIAA1217, the human homolog of murine sickle tail, is involved in the development of intervertebral discs (59, 60) and has been implicated with cancer progressions by inducing epithelial-mesenchymal transition (61, 62).

**Fig. 5:**
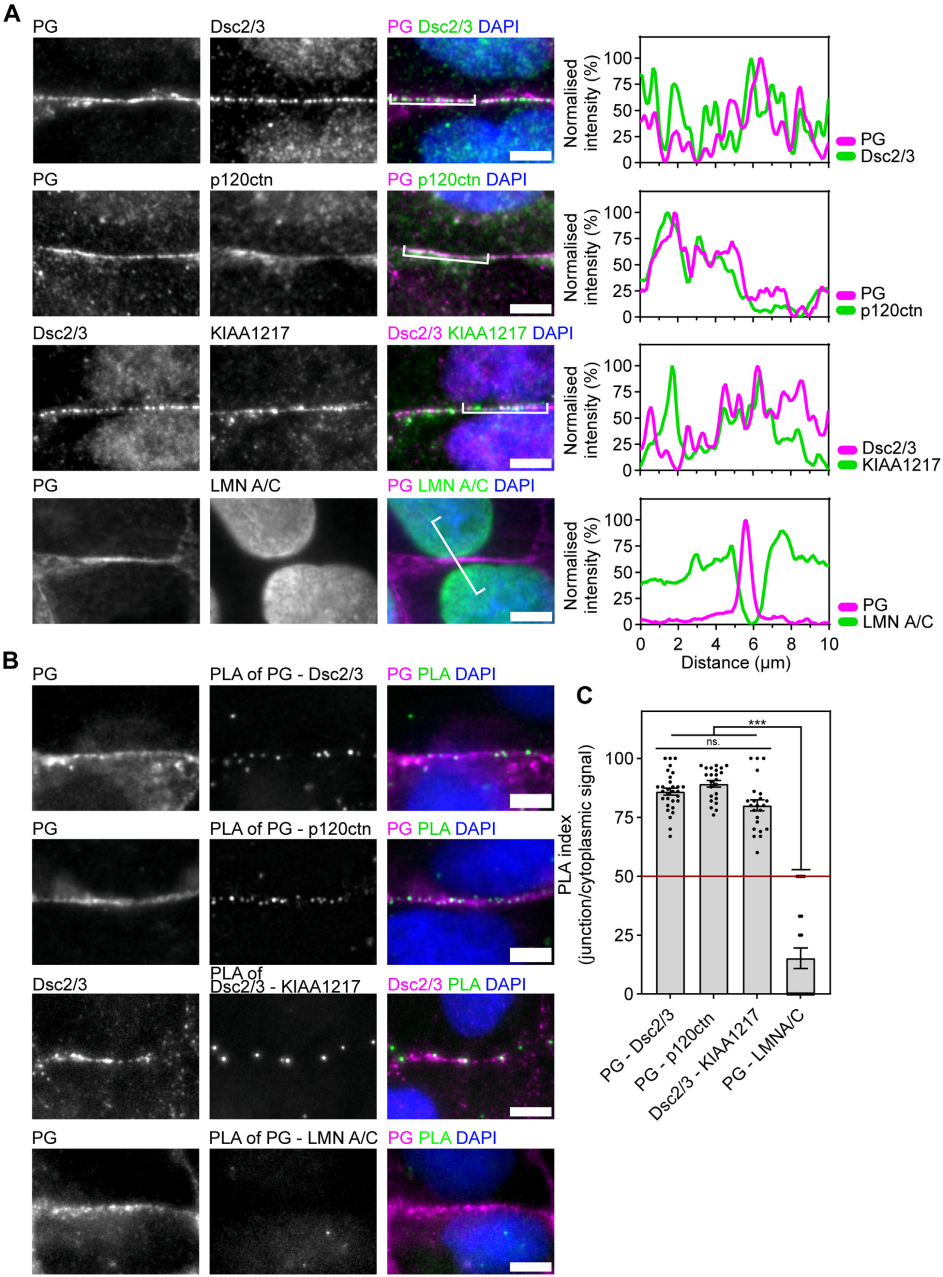
***In situ* interaction of p120 catenin and KIAA1217 with desmosomes.***A*, immunofluorescent staining in wt MDCK cells. The desmosomal proteins plakoglobin (PG) and desmocollin 2 and 3 (Dsc2/3) were used as a positive control. The nuclear protein lamin A/C (LMNA/C) was used as a negative control. Novel interaction partners, p120 catenin, and KIAA1217 are shown. The intensity profiles correspond to the *white line* in the merged images. Representative of three repeats. *B*, *in situ* proximity ligation further confirms the close interaction of p120 catenin and KIAA1217 with desmosomes. The scale bars represents 5 μm. *C*, the ratio of PLA signals at cell–cell junctions and cytoplasm (*N* = 3), ns. = no significant difference, ∗∗∗*p* < 0.0001 (Kruskal–Wallis with Dunn’s multiple comparisons test). MDCK, Madin–Darby canine kidney II cell; PLA, proximity ligation assay.

We have performed colocalization staining and *in situ* PLAs (33, 63)) to validate two of the novel protein interactions at desmosomes in wt MDCK cells (Fig. 5). The nuclear protein Lamin A/C was used as a negative control. Lamin A/C, identified but not statistically enriched in all of the desmosomal proximitomes, showed no colocalization and negligible PLA signal (PXD037933 ProteomeXchange Consortium, Fig. 5). p120 catenin, which associates with desmogleins during desmosome assembly (64), colocalized with both PG and DP (Fig. 5*A* and Supplemental Fig. S5) and showed high PLA signal similar to the positive control of PG and Dsc2/3 (Fig. 5*B*). Interestingly, KIAA1217, predicted to be cytoplasmic (59), showed colocalization with desmosomes (Dsc2/3) and high PLA signal and cell–cell junctions (Fig. 5). The role of the novel interaction partners in the regulation and signaling capacity of desmosomes remains to be determined.

Known interaction partners of desmosomes, Crk and CrkL, were significantly enriched irrespective of the adhesion state of desmosomes. Both Crk and CrkL were shown to localize to desmosomes and to be critical for desmosome integrity, in particular for the localization of PG to the desmosomal plaque in the epidermis of Crk/CrkL KO mice (50). These proteins were overrepresented in cell–cell adhesion-relevant GO terms “cadherin binding” and “molecular adaptor activity” and outline the proximity with potential signaling crosstalk to the neighboring cadherin- and nectin-associated AJs (65, 66, 67, 68).

A novel association appeared in Cluster 9 that hinted at some crosstalk of desmosomes with tight junctions. This cluster depicted mainly Dsc2a∗-associated constant proteins and included Coxsackie virus and adenovirus receptor that is necessary for tight junction integrity and transepithelial leukocyte migration (Fig. 4*A* and Supplemental Fig. S4) (69, 70). Coxsackie virus and adenovirus receptor is also a receptor for Coxsackievirus B3 and its proximity to DCs may be relevant for cardiomyocyte biology. A recent study showed that Coxsackievirus B3 promotes viral myocarditis through the disruption of desmosomes by cleaving Dsg2 and Dsc2 and subsequent promotion of WNT/β-catenin signaling by PG degradation (71). Cluster 9 also included NCK adaptor protein 1 (NCK1), which is involved in signal transduction from receptor tyrosine kinases and the actin cytoskeleton (72, 73). It should be noted that, unlike desmosomes, the majority of the adherens and tight junction proteins are linked to the actin cytoskeleton. In contrast to the IFs that are associated with desmosomes, the actin cytoskeleton contains motor proteins that mediate contractility. The proximity of the two systems may be consistent with some of the actin-dependent mechanisms that regulate desmosome function, including the actin-dependent internalization of desmosomes (11, 25).

A group of novel proteins in cluster 10 that remained in the proximitome of desmosomes irrespective of the adhesion state belonged to terms associated with DNA and histone binding (“histone binding”, “four-way junction DNA binding,” and “DNA binding, bending”) (Fig. 4*B*), including the high mobility group proteins 1 to 3 (HMGB 1–3) and the ANP 32 family members A, B, D, and E (ANP32 A/B/D/E). Network analysis of cluster 10 including computational STRING-based evidence scores revealed the strong relation of adhesion-relevant proteins with desmosomal components and the lesser-known relationship of this subnetwork to the nuclear proteins (Fig. 4*D*). These data suggest a potentially important yet rather unexplored functional role of desmosomes in the regulation of transcription factors. HMGB1/2/3 are drivers of various cancers and have nuclear chromatin binding functions, regulation of both telomerase activity and transcription, as well as cytoplasmic and cytokine-like functions, driving inflammation, and proliferation when released from cells (74, 75, 76). Furthermore, HMGB proteins were shown to be regulated by both PG and β-catenin (β-catenin/WNT pathway) (75). Additionally, considering their cytokine-like functions, they may play a pivotal role in regulating cell proliferation in relation to IF-associated cell junctions. It is interesting to speculate that PG is also directly involved in the regulation of HMGB1/2/3 providing a reciprocal signal of the adhesion state of desmosomes to the nucleus. Alternatively, PG might compete with β-catenin in the regulation of HMGBs. It should be noted that, whilst the HMBGs are “constant” proteins, their prey-to-bait ratios are substantially greater during the Ca^2+^-dependent phase than during hyper-adhesion (Fig. 4*C*) so it may be that their functional significance is greater during keratinocyte activation (*e.g.*, wound healing (77)) and declines as desmosomes mature.

In contrast, erbin is a constant protein that shows an increased prey/bait ratio in hyper-adhesion. Intriguingly, erbin was the most enriched protein within the PG∗ datasets during hyper-adhesion and was also increasingly enriched in proximity to Dsc2a∗ and Pkp2a∗ (Fig. 4, *A* and *C*, and Supplemental Fig. S3*C*). Erbin is an adaptor protein and a critical regulator of the localization and signaling of the receptor tyrosine-protein kinase erbB-2 (ERBB2/HER2) and Ras-mediated MAPKs signaling pathways (78). It has also been associated with numerous other signaling pathways and several binding partners have been identified including the desmosomal accessory protein Pkp4 (also known as p0071) (79). Interestingly Pkp2, 3 and 4 follow a similar increased presence in the proximitome as erbin. Since the interaction of erbin and Dsg1 was shown to disrupt SHOC2–Ras–Raf complexes (51), the neighborhood could play a critical role in signaling cell quiescence as cell density increases and desmosomes progressively switch to a hyper-adhesive state.

### Ca^2+^ Dependence: Possible Roles in Desmosome Assembly

The Ca^2+^-dependent phase is associated with desmosome assembly, a process which appears to continue for at least 36 h subsequent to its initiation in MDCK cells (80). Desmosomes also become Ca^2+^-dependent upon epithelial wounding (81) and during wound healing, in areas where keratinocytes become activated for migration that requires motility and desmosome turnover. At this time desmosomes are less stable than during hyper-adhesion (8, 17, 25, 81) and both assembly and internalization require crosstalk with the actomyosin machinery (14, 25, 66).

Such crosstalk is reflected particularly in the group of prey hits of PG∗ that were enriched in Ca^2+^-dependent conditions (cluster 11 in Fig. 4*A*; Supplemental Figs. S3, *B* and *C* and S4) and were mostly linked to actin-associated terms (“actin-dependent ATPase activity,” “actin filament binding,” “actin binding,” and “microfilament motor activity”) (Fig. 4*B*; cytoskeleton in Fig. 5). Members of these groups were the actin-based motor proteins myosin 1B and E, actin-plasma membrane cross-linker moesin, Arp2/3 complex subunit actin-related protein 3 (*i.e.*, ARP3) and the IQ calmodulin-binding motif containing GTPase-activating protein 1, all of which are known regulators of the actin cytoskeleton (82, 83, 84, 85, 86).

Another large fraction of Dsc2a∗ proximal interactors (cluster five in Fig. 4*A* and Supplemental Fig. S4) fall into similar categories as actin regulatory proteins and contain the Rho GTPases and related proteins, such as Cdc42 effector protein 4, RAS-related, and p21- (RAC1) activated kinase 2. All these prominent actin modulators are associated with membranes and possibly have a role in the concomitant assembly of desmosomes and AJs (87). In line with the notion that desmosomes increasingly separate from other junctions (see above) during maturation is the finding that other junctional receptors such as integrins (INGB1), claudins, (CLDN1), and EpCAM (EPCAM) are exclusively present in the proximitome of Dsc2a under Ca^2+^-dependent conditions.

**Fig. 6:**
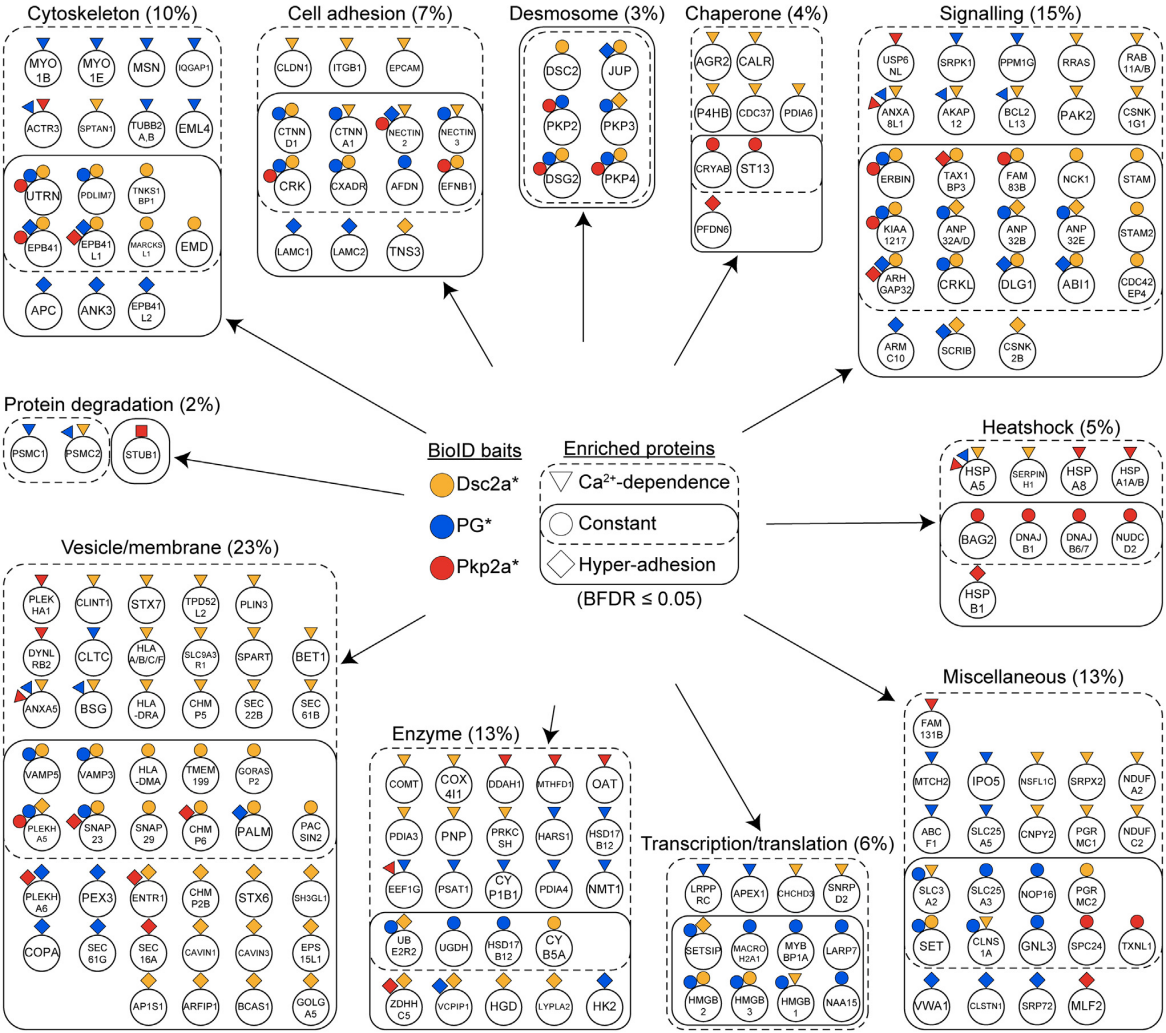
**Functional characterisation of desmosome proximal proteins.** The function of the 189 proximal proteins (BDRF ≤0.05) of BirA-myc–tagged desmocollin 2a (Dsc2a∗), plakoglobin (PG∗; merged data plakoglobin-BirA-myc and myc-BirA-plakoglobin data), or plakophilin 2a (Pkp2a∗) were annotated based on primary literature and the Human Protein Atlas and classified into the indicated categories. The proximal baits are represented on each prey with the corresponding coloured geometric shapes (Dsc2a∗ in *yellow*, PG∗ in *blue*, and Pkp2a∗ in *red*). Boundaries and *geometric shapes* indicate whether the prey proteins were significantly enriched in data curated from MDCK cells cultured confluently for either 1 day and thus with Ca^2+^-dependent desmosomes (*dotted line, triangular bait*), for 5 days and thus hyper-adhesive desmosomes (*solid line, square bait*) or enriched in both culture conditions (*overlap, circular bait*). The percentages of the total protein number are indicated in *brackets*. BFDR, Bayesian false discovery rate; MDCK, Madin–Darby canine kidney II cell.

A smaller fraction of proteins in Ca^2+^-dependent desmosomes was uniquely linked to Pkp2a (cluster 2 in Fig. 4*A*; Supplemental Fig. S4), most of which belong to HSPs (compare with Fig. 6). Amongst them were family members of Hsp70 proteins (HSPA1A, HASPA1B, HSPA5, and HSPA8). Gao and Newton showed that Hsp70 is involved in the stabilization and rephosphorylation of PKCs (88). These results are consistent with the importance of PKCs in the regulation of the desmosomal adhesion state (8, 10, 13, 81, 89). Translocation of PKCα to desmosomes and phosphorylation of DP was shown to precede Ca^2+^ dependence of desmosomes (8, 10). Furthermore, PKC-scaffolding annexins were present particularly under Ca^2+^-dependent conditions for all baits (cluster 5 in Fig 4*A*: Supplemental Fig. S4). Annexins are known substrates and scaffolding proteins of multiple PKC isoforms (90), so their juxtaposition to desmosomes (ANXA5, vesicle/membrane fraction, Fig. 6) might contribute to the PKCα-mediated adhesion switch of desmosomes shown by Wallis *et al.* (8).

One of the largest fractions uniquely associated with different adhesion states is linked to vesicles and membranes, predominantly in the neighborhood of the transmembrane protein Dsc2a (clusters 5 and 8 in Fig. 4*A*; Supplemental Fig. S4; Fig. 6). Many of these proteins are involved in membrane organization, transport of vesicles or membrane channels. How the dramatic switch in membrane neighborhood contributes to desmosome function or, vice versa, how desmosomes may contribute to the changes of this subcellular compartment, with a potentially strong impact on cellular function, remains to be elucidated.

### Hyper-Adhesion: Functions of Mature Desmosomes

Hyper-adhesion is the condition of most desmosomes in tissues, and thus the interacting proteins found here most probably represent the stable state of desmosomal function. The current analysis reveals several novel interactions of desmosomes as well as some confirmatory indications.

Cluster 8 included Dsc2a∗ preys uniquely enriched under hyper-adhesive conditions, many of which are linked to the localization of membrane-associated proteins (Fig. 4*A* and Supplemental Fig. S4). Those included proteins involved in caveolar endocytosis including caveolae-associated proteins 1 and 3 (Cavin1/3) and epidermal growth factor receptor pathway substrate 15 like 1 (91, 92). These results are consistent with results from Brennan *et al.* showing colocalization of Dsg2 with Cavin1 (93). In their study, authors proposed a caveolae-dependent internalization and subsequent degradation of proteolytically truncated Dsg2, critical for the maintenance, and stability of desmosomes. Our results suggest that this process is important for matured hyper-adhesive desmosomes.

Clusters 4 and 6, and to some extent cluster 7, represent particularly PG∗ preys enriched in hyper-adhesion (Fig. 4*A*; Supplemental Figs. S3 and S4). Calsyntenin-1, alone forming cluster 6, was discovered in postsynaptic membranes and belongs to the superfamily of cadherins, thus potentially containing a PG binding site (94). The tumor suppressor adenomatous polyposis coli (APC) protein appears in the vicinity of PG (cluster 7; Supplemental Fig. S4). APC was previously found to bind PG, and together with the scaffolding protein axin, it is possibly involved in the degradation and thus the homeostasis of PG levels of mature desmosomes (95). Cluster 4 (Supplemental Fig. S4) included the scaffolding proteins ankyrin 3 (ANK3 also known as Ankyrin G) and erythrocyte membrane protein band 4.1 like 2. These are part of the cortical spectrin-actin networks as they are both able to bind spectrin (96). The importance of an intact cortical actin network for the turnover and stability of the desmosomal complex was recently shown by knockout and knockdown of α-adducin, a component of the spectrin-actin network, in keratinocytes (21, 97). Our results appear consistent with the view that the development of desmosomal hyper-adhesion is part of a process of stabilization of the cortical cytoskeleton and the quiescence of cells, also involving spectrin and associated proteins (98, 99).

### Recategorization of Protein Function

GO analysis allows rapid insight into potential cellular and molecular functions of preys based on a vast array of experimental data. However, since GO annotations continuously evolve as experimental data become available (100, 101), GO enrichment analysis can be affected by annotation bias (annotations from only a few well-studied genes) or literature bias (a few articles that contribute disproportionally to annotations). Such bias may explain the relatively high number of neurology-related GO terms even though our data relate to epithelial cells. Examples of such terms include “postsynaptic density” and “asymmetric synapse” (Fig. 3*A*). Proteins with these annotations include p120ctn, nectin 3, and the Rho GTPase-activating protein 32 (ARHGAP32 *i.e.*, RICS). A recent preprint by Li *et al.* shows that ARHGAP32 is critical for the assembly and organization of desmosomes through its direct interaction with DP and the regulation of actomyosin contractility (102). However, the GO annotation for ARHGAP32 is currently based on studies showing its involvement in the β-catenin-N-cadherin receptor signaling and synaptic adhesions of neurons (103).

To minimize such bias we complemented the GO analysis with our own map of manually annotated protein functional terms based on “The Human Protein Atlas,” an omic database that provides a wider range of information. Ten functional categories, which encompassed all preys, evolved during the process of manual annotation: cytoskeleton, cell adhesion, desmosome, chaperone, signaling, heat shock, transcription/translation, enzyme, vesicle/membrane, protein degradation, and miscellaneous when multiple categories applied or the protein function was unknown (Fig. 6). While this complementary functional classification does not comprise a statistical “enrichment analysis” and could be extended in further subgroups, it highlights functional terms that may be more comprehensive in relation to epithelial cell functions. Figure 6 also clearly illustrates the changes in the environments of these major desmosomal proteins during the process of maturation from Ca^2+^ dependence to hyper-adhesion.

## Conclusion

**Fig. 7:**
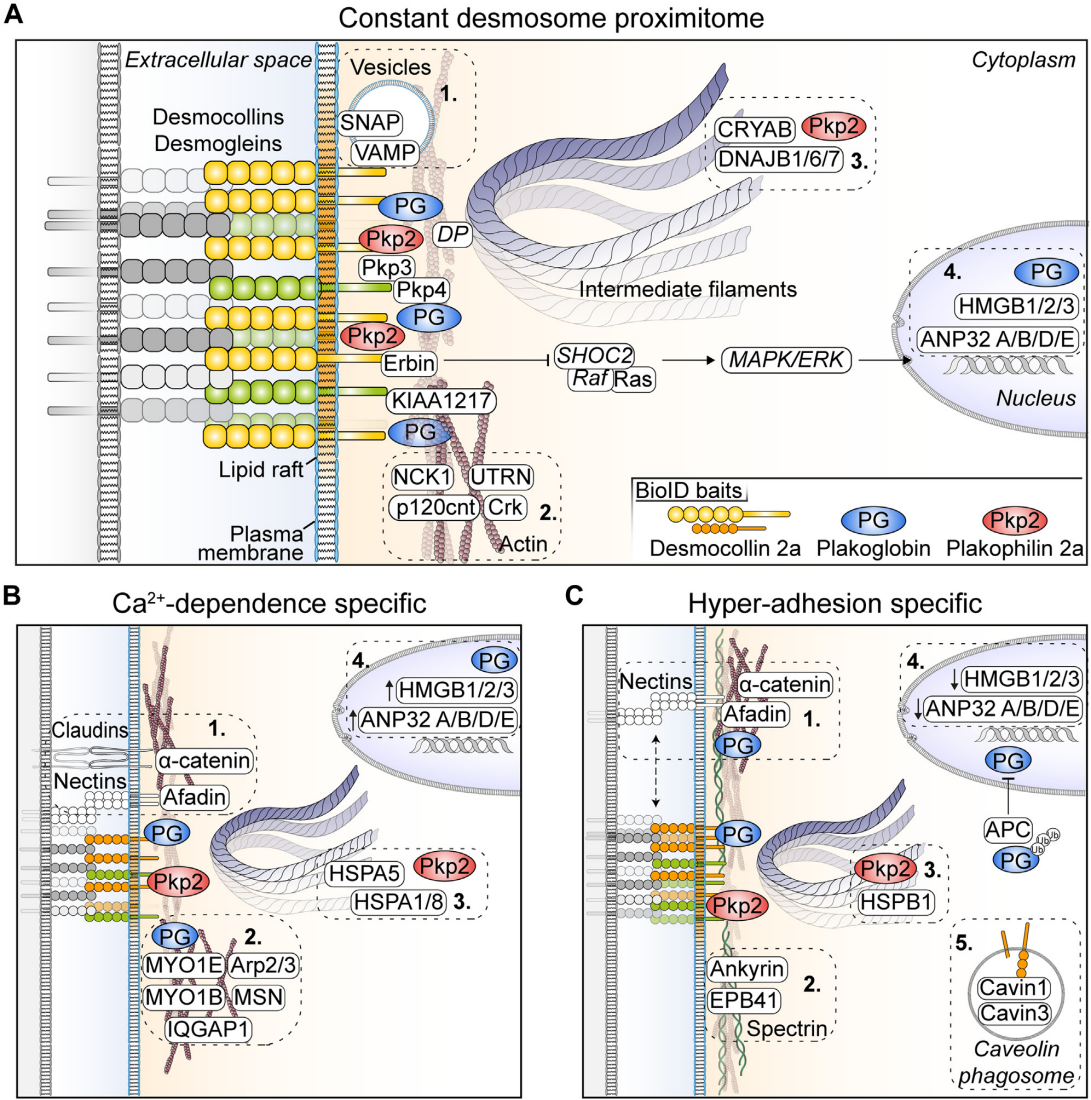
**Schematic diagrams of the desmosome proximitome and differences between the Ca**^**2+**^**-dependent and hyper-adhesive phases.***A*, constant proteins that are present in both phases, (*B*) proteins present exclusively during Ca^2+^ dependence, (*C*) proteins present exclusively during hyper-adhesion. Not all significantly enriched proximal proteins are shown. Those written in *italics* were not significantly enriched but have been inserted for discussion purposes. For further details see text where the *numbered boxes* are referred to. (*Small solid arrows* indicate increased or decreased protein ratios).

The picture that emerges is of a junction which, though fundamentally stable and mechanical in function, possesses extensive signaling capacity and which changes its interactome considerably according to whether it is in a less stable, immature state or a more stable, more strongly adhesive mature state. Furthermore, our data support our recently proposed concept of desmosomal dualism as the characteristics observed here revealed of Pkp2a, the constantly dynamic component, differ from those of Dsc2a and PG, which are much more stable (25). In Figure 7, we summarize this and emphasize some of the major points that emerge from our results.

During junction assembly, desmosomal proteins are closely associated with those of AJ but subsequently they segregate into separate junctions. This compartmentalization of junctions is clearly indicated in our findings where the AJ proteins nectin 2 and 3 and α-catenin are close to Dsc2a when desmosomes are Ca^2+^-dependent (box 1. in Fig. 7*B*) but become spatially more distinct as desmosomes mature to hyper-adhesion (box 1. in Fig. 7*C*). These proteins, however, retain proximity to PG, possibly reflecting the capacity of PG to locate at AJs as well as desmosomes (66). This highlights the fact that both PG and Pkp2 can reside in nondesmosomal locations.

Spatial separation of junctions is accompanied in desmosomes by intriguing changes in the associated actin-binding proteins. Different actin-binding proteins interact, especially with PG, depending on the adhesion state (box 2 in Fig. 7*B*; boxes 1 and 2 in Fig. 7*C*). These include adaptor proteins (*e.g.*, p120 ctn, α-catenin, and afadin), and those involved in endocytosis (*e.g.*, myosin 1E) or in actin dynamics (*e.g.*, NCK1 and Arp2/3). A shift toward cortical spectrin-associated proteins (*e.g.*, ankyrin and erythrocyte membrane protein band 4.1) suggests a remodeling and stabilization of the cortical spectrin-actin network concomitant with the maturation of desmosomes box 2 in Fig. 7*C*). We also noted the lack of interaction of the Dsc2a, PG, and Pkp2a baits with keratin proteins. However, the HSPs CRYAB and HSPB1, which associate with Pkp2a as constant and hyper-adhesion proteins, respectively, have been shown elsewhere to interact with IFs, possibly preventing their aggregation (104, 105). Also noteworthy is that these two Pkp2-associated HSPs change as desmosomes mature (boxes 3 in Fig. 7, *B* and C). It is interesting to speculate whether this may indicate a Pkp2a-regulated change in the nature of keratin filament interaction with desmosomes or whether it represents a separate interaction between Pkp2a and keratin filaments in a location remote from desmosomes as shown in Figure 7.

The DC desmoglein 1 has been shown to regulate the Ras/MAPK/ERK pathway and thus transcriptional activity in epidermal keratinocytes (51). In view of this, we speculate that the ratios of erbin increasing near hyper-adhesive desmosomes may indicate that desmosomes regulate cell quiescence *via* the same pathway (Fig. 7*A*, italics). The association of other nuclear proteins with desmosomes, in ratios decreasing with hyper-adhesion (Box 4 in Fig. 7), suggests further potential for regulation of gene expression by desmosomal proteins. Whether this may involve an extradesmosomal role for PG or some type of cell junction to nucleus signaling as has been found for tight junction remains to be seen (106). Proximity to APC may potentially reduce the cytoplasmic and nuclear levels of PG and thus affect desmosome-related signaling (95, 107, 108).

Desmosomes are embedded in lipid rafts, which are microdomains of the plasma membrane enriched in sphingolipids and cholesterol and which are believed to play a role in desmosome assembly, function, and disassembly (109, 110). In this context, it is interesting that several membrane and vesicle-associated proteins are constantly in proximity to Dsc2a (box 1. in Fig. 7*A*). In addition the proximity of cavin 1 and 3 to Dsc2a (box 5 in Fig. 7*C*) suggests homeostasis of DCs through caveolae-dependent internalization is important in hyper-adhesive desmosomes. Desmosomes are remarkably stable structures (25, 111), with FRAP studies, indicating no more than a 20% turnover of Dsc2a within 5 min. However, the appearance of cavins in our present studies following biotin labeling for 12 h indicates a possible continuous turnover of Dsc2a throughout the lifetime of the desmosome.

In closing, we would like to stress our belief in the importance of maturation studies such as presented here. Though currently limited, the evidence suggests that the hyper-adhesive state acquired by desmosomes during extended confluent tissue culture resembles that acquired by and maintained by desmosomes in tissues (7, 8, 9, 13) and is therefore highly relevant to the function of desmosomes *in vivo*. One of our major findings has been the substantial differences between the proximities of desmosomes in this hyper-adhesive state compared to those that are newly formed.

## Data Availability

The MS proteomics raw and processed data have been deposited to the ProteomeXchange Consortium (http://proteomecentral.proteomexchange.org) *via* the PRIDE partner repository with the dataset identifier PXD037933.

Username: reviewer_pxd037933@ebi.ac.uk

Password: 1LR4c7jj

## Supplemental data

This article contains supplemental data (50, 52).

## Conflict of interest

The authors declare no competing interests.
