# Supplementary material for: Proximity Mapping of Desmosomes Reveals a Striking Shift in Their Molecular Neighborhood Associated With Maturation
Source: Mol Cell Proteomics. 2024 Feb 10;23(3):100735. doi: 10.1016/j.mcpro.2024.100735 (PMC10943070; doi:10.1016/j.mcpro.2024.100735)
Supplement: Supplemental Data [file mmc1.docx]

**Supplemental Information**

Table S1. **List of desmosomal proximitome of Ca^2+^-dependent desmosomes.** List of enriched prey proteins (high-confidence cut-off of BFDR ≤ 0.05) identified by BirA-myc tagged desmosomal constructs, desmocollin 2a (Dsc2a), plakoglobin-BirA-myc (PG-C), myc-BirA-plakoglobin (PG-N) and myc-BirA-plakophilin 2a (Pkp2a). Proximal proteins were identified in samples of MDCK cells cultured confluently for 1 day and thus with Ca^2+^-dependent desmosomes. Prey and BirA* control intensities, fold changes (average LFQ intensities of test interaction divided by the average in controls i.e. bait/BirA* control) and BFDR (Bayesian false discovery rate) were calculated using SAINTexpress from LFQ ion intensities generated by MaxQuant. A BFDR of 0.05 which most probably represents true proximity interactions was used as a threshold. If preys are classified as ribosomal proteins and whether they were identified by BioID with desmoplakin or E-cadherin are given.

Table S2. **List of desmosomal proximitome of hyper-adhesive desmosomes.** List of enriched prey proteins (high-confidence cut-off of BFDR ≤ 0.05) identified by BirA-myc tagged desmosomal constructs, desmocollin 2a (Dsc2a), plakoglobin-BirA-myc (PG-C), myc-BirA-plakoglobin (PG-N) and myc-BirA-plakophilin 2a (Pkp2a). Proximal proteins were identified in samples of MDCK cells cultured confluently for 5 days and thus with hyper-adhesive desmosomes. Prey and BirA* control intensities, fold changes (average LFQ intensities of test interaction divided by the average in controls i.e. bait/BirA* control) and BFDR (Bayesian false discovery rate) were calculated using SAINTexpress from LFQ ion intensities generated by MaxQuant. A BFDR of 0.05 which most probably represents true proximity interactions was used as a threshold. If preys are classified as ribosomal proteins and whether they were identified by BioID with desmoplakin or E-cadherin are given.

Table S3. **Overview of the desmosomal proximitome with functional annotation.** List of enriched prey proteins (high-confidence cut-off of BFDR ≤ 0.05) identified by BirA-myc tagged desmosomal constructs, desmocollin 2a (Dsc2a), plakoglobin-BirA-myc (PG-C), myc-BirA-plakoglobin (PG-N) and myc-BirA-plakophilin 2a (Pkp2a). Proximal proteins were identified in samples of MDCK cells cultured confluently for 1 day and thus with Ca^2+^-dependent desmosomes (Ca^2+^-dep.) or for 5 days and thus with hyper-adhesive desmosomes (hyper-adh.). The gene names of human orthologs of the preys, their protein names and the functional annotation of the preys (manual annotation based on the primary literature and the Human Protein Atlas) are given. The list is sorted according to the enrichment with the baits, the adhesion state and the average fold change over the BirA-myc control samples.

Table S4. **Ontological analysis of desmosomal proximitomes.** Supporting table for Figure 3 and S2B. Gene ontology (GO) enrichment analyses of all significantly enriched prey proteins (BFDR ≤ 0.05) were performed using the R package ClusterProfiler against human GO annotations including molecular function (MF), cellular component (CC) and biological process (BP). The BioID data sets of the distinct desmosomal baits, Dsc2a* (desmocollin 2a-BirA-myc), Pkp2a* (myc-BirA-plakophilin 2a) and PG* (combined data of myc-BirA-plakoglobin and plakoglobin-BirA-myc) are indicated in the first column, followed by the GO category (MF, CC or BP), the specific GO ID and its description. The prey proteins with respective GO annotations are listed in column E. The GeneRatio indicates the ratio of input genes (preys) that are annotated with the GO term and BgRatio indicates the ratio of all genes that are annotated with this term. The p-value and the adjusted p-value (corrected for multiple testing using the Benjamini-Hochberg method) are given. The significance of the Clusterprofiler results was assessed using the adjusted p-value.

Table S5. **List of primers.** All primers that were used to clone desmosomal BioID constructs.

**Figure S1.**

**
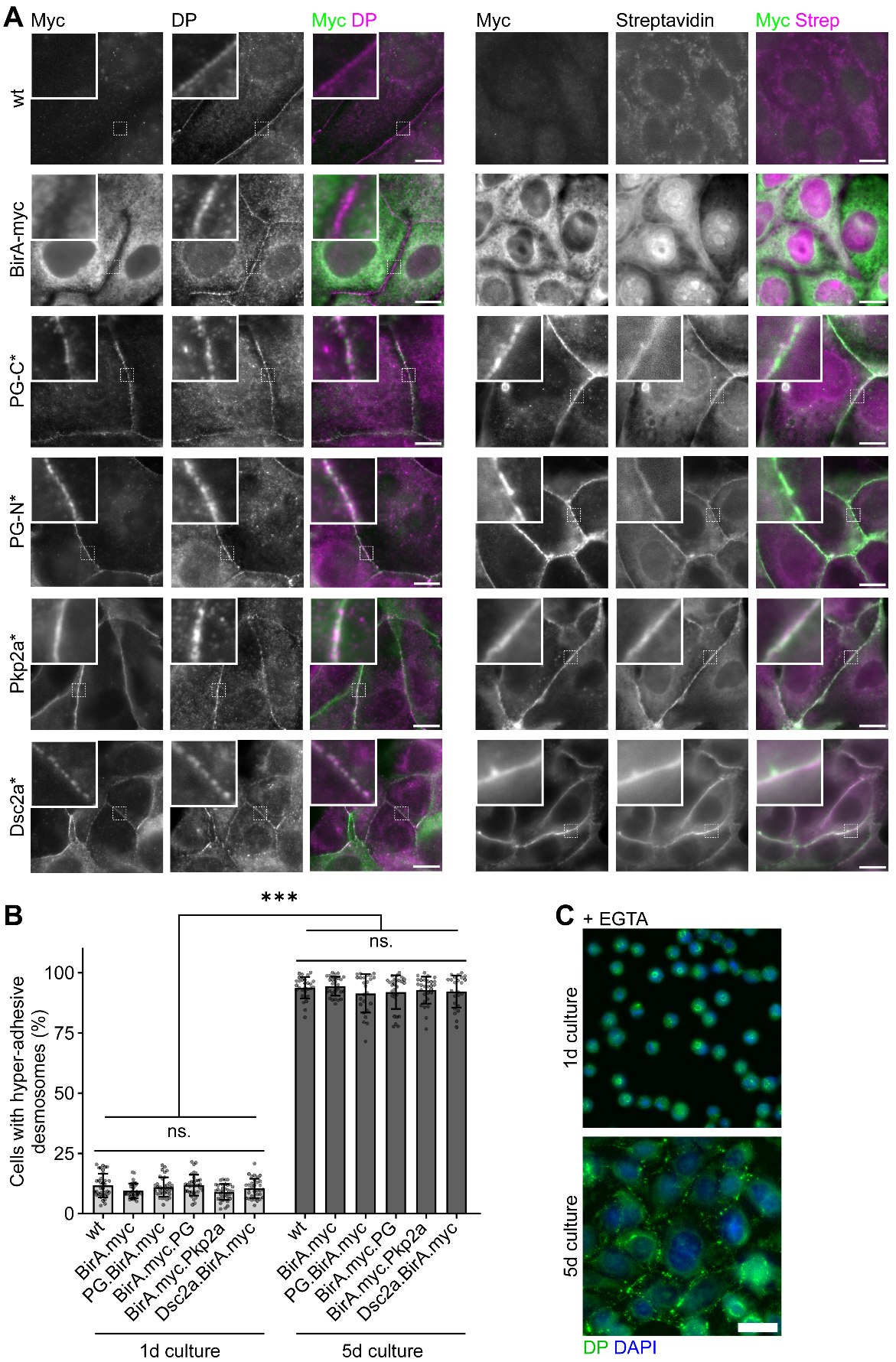
**

Figure S1. **Localisation of BioID constructs to desmosomes and acquisition of hyper-adhesion of stable cell lines. (A)** Confocal microscopy images of MDCK cells stably expressing BioID constructs as shown in Figure 1 and the parental wild-type cells. Cells were cultured subconfluent for 24 h including 16 h of incubation with 100 µM biotin before immuno-labelling desmoplakin (DP) (left panel) or myc and fluorescently conjugated streptavidin (right panel). Data representative of three biological repeats. Scale bars: 10 µm. **(B)** Quantification of cells with hyper-adhesive desmosomes. The cells were cultured confluent for 1 or 5 days, treated with Ca^2+^-chelating medium for 90 min and stained for desmoplakin. N=3 **(C)** Representative images of desmosomes of MDCK cells acquiring hyper-adhesion. Scale bar: 20 µm.

**Figure S2.**


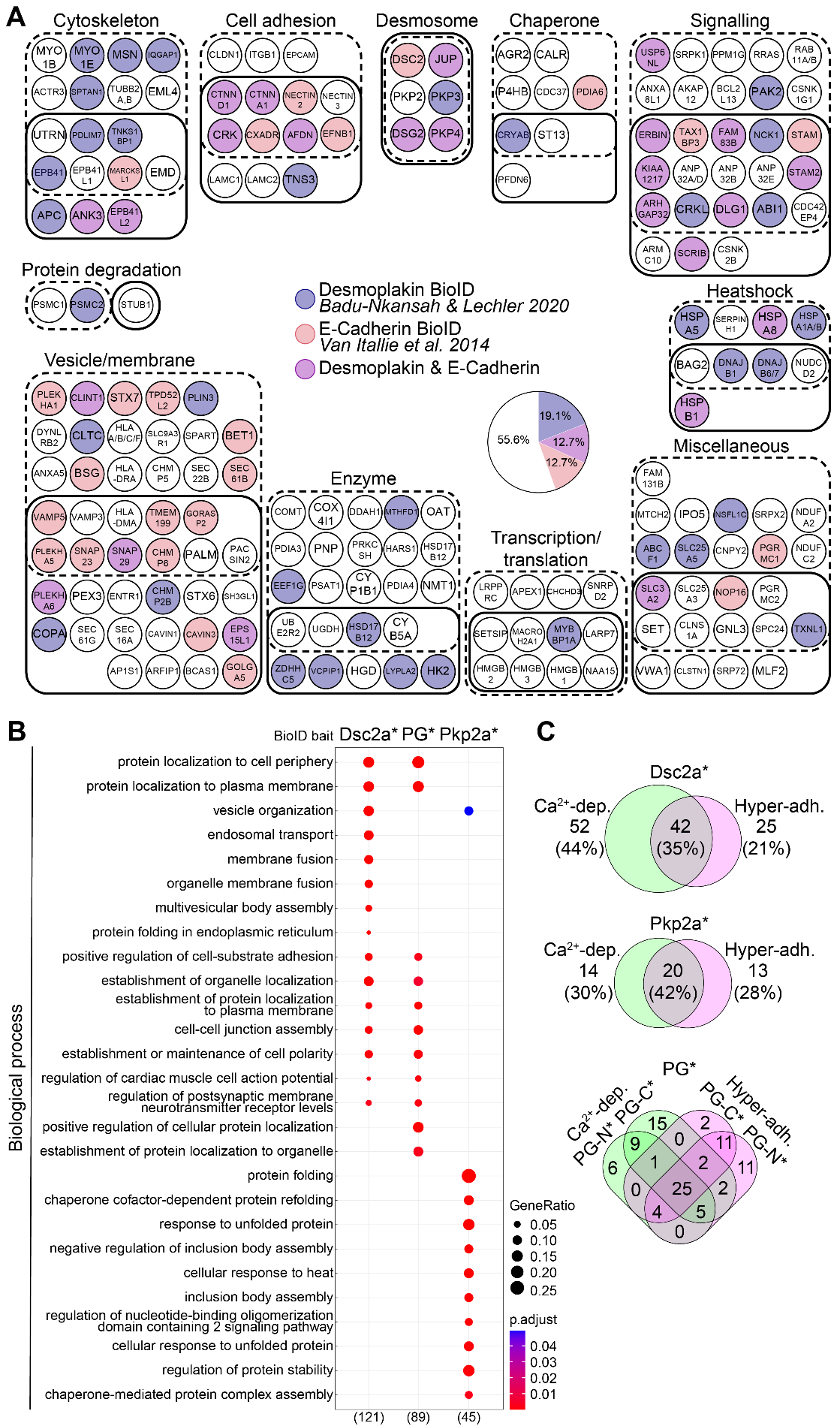


Figure S2. **Further analysis of the desmosome proximitome.** Accompanies Figure 2 and 3**. (A)** Functional network of desmosome BioID data presented in this study (combined proximal proteins to desmocollin 2a-BirA-myc (Dsc2a*), myc-BirA-plakoglobin (PG-N*), plakoglobin-BirA-myc (PG-C*), myc-BirA-plakophilin 2a (Pkp2a*); BFDR ≤ 0.05) compared to BioID studies of desmoplakin and E-cadherin (Badu-Nkansah and Lechler, 2020; Van Itallie et al., 2014). Proteins were annotated using the primary literature and the Human Protein Atlas and classified into the indicated categories (see text relating to Figure 6). Boundaries indicate whether the prey proteins were significantly enriched in data curated from MDCK cells cultured confluently for either 1 day and thus with Ca^2+^-dependent desmosomes (dotted line) or for 5 days and thus hyper-adhesive desmosomes (solid line). Proximal prey proteins are coloured in purple when they were also identified in the desmoplakin interactome, pink when they were present in the E-cadherin interactome and magenta when they were present in both the desmoplakin and E-cadherin datasets. The pie chart shows the overlapping prey proteins identified in this study in percentage. **(B)** GO enrichment analysis of the 189 proteins partial desmosomal proximitome. The top 10 overrepresented terms of each bait (Dsc2a*, Pkp2a* and PG* [PG-N* and PG-C* combined]) under the biological process category are shown. The number of annotated proteins is shown in brackets. (Note 7 protein hits encompassed protein groups of which all members were included for GO analysis because we could not distinguish between different isoforms. For details see Materials and Methods.) p.adjust, adjusted P value. GeneRatio, the proportion of total proteins identified in each GO term. **(C)** Venn diagrams illustrating the number of overlapping proteins depending on the bait and adhesion state.

**Figure S3.**


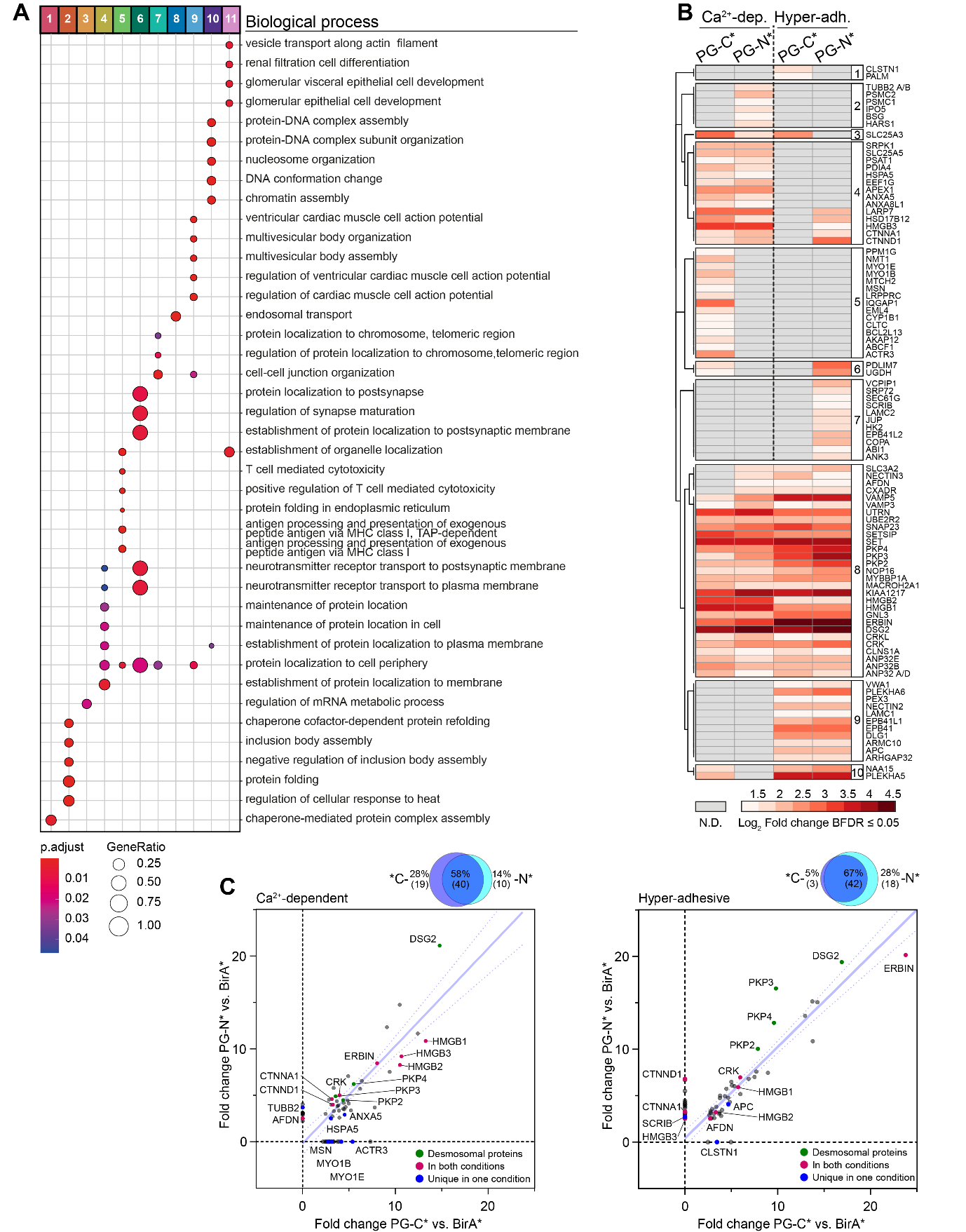


Figure S3. **Functional enrichment analysis of prey** **clusters and hierarchical clustering of preys of plakoglobin**. Accompanies figure 4. **(A)** GO analysis of the prey clusters identified from hierarchical clustering of the desmosomal proximitome (Fig. 4 A). The top ten overrepresented GO terms under the biological process category are shown. p.adjust, adjusted P value. GeneRatio, the proportion of total proteins identified in each GO term. **(B)** Hierarchical clustering was performed on the proteins identified in the plakoglobin (PG) BioID proximitome and the results are displayed as a heatmap. **(C)** Scatter plot and area-proportional Venn diagram showing the relationship of PG-N* and PG-C* prey proteins in either Ca^2+^-dependent or hyper-adhesive conditions (fold change enrichment over BirA*). The 95% confidence interval of the regression line is displayed as a dotted confidence band.

**Figure S4.**


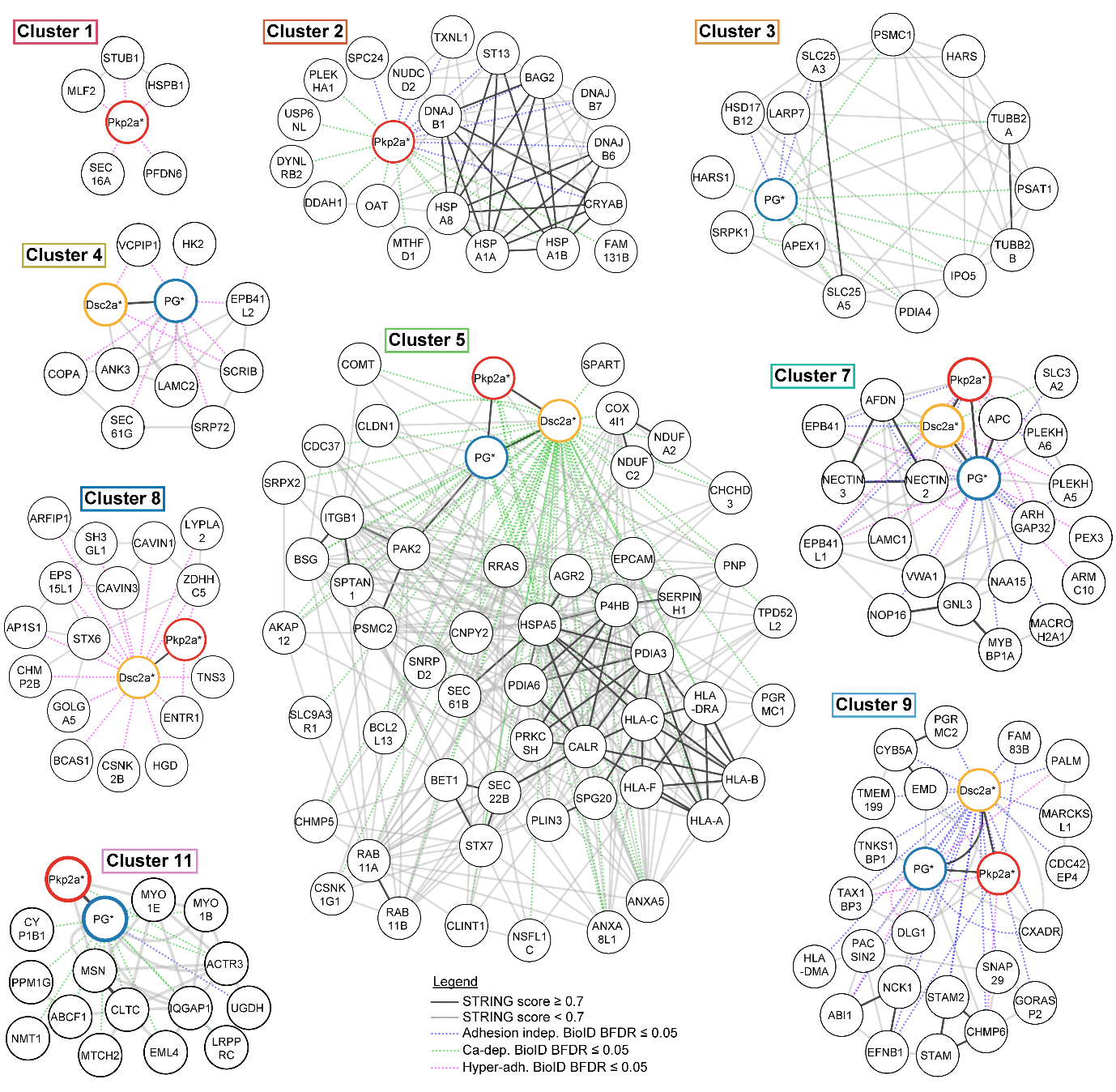


Figure S4. **Network analysis of prey clusters.** STRING network analysis of the prey clusters identified from hierarchical clustering of the desmosomal proximitome (Fig. 4). Nodes of bait proteins are colour codes with Pkp2a* in red, Dsc2a* in yellow and PG* (merged PG-N* and PG-C*) in blue. Edges indicate protein-protein interactions: solid dark grey lines indicate a STRING score ≥ 0.7; solid light grey lines a STRING score below 0.7 and dotted lines BioID proximity with a BDFR ≤ 0.05 presented in this study (blue = adhesion independent, green = Ca^2+^-dependent, magenta = hyper-adhesive). Cluster 10 can be found in Figure 4D and Cluster 6 only contains Calsyntenin 1 which was only enriched with PG-C* under hyper-adhesive conditions.

**Figure S5.**


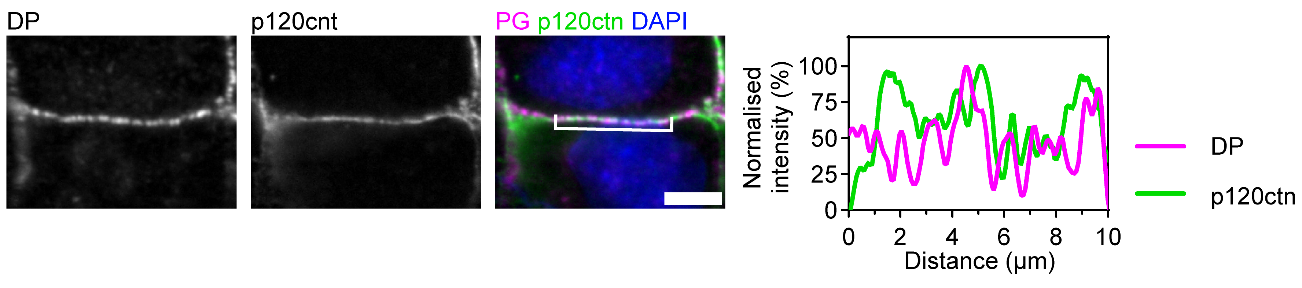


Figure S5. **p120 catenin colocalises with desmoplakin.** Immunofluorescent staining against p120 catenin and desmoplakin in wt MDCK cells with Ca^2+^-dependent desmosomes. The intensity profile corresponds to the white line in the merged image. Representative of three repeats. Scale bar: 5 µm.
